# A compendium of temperature responses of Rubisco kinetic traits: variability among and within photosynthetic groups and impacts on photosynthesis modeling

**DOI:** 10.1093/jxb/erw267

**Published:** 2016-07-12

**Authors:** Jeroni Galmés, Carmen Hermida-Carrera, Lauri Laanisto, Ülo Niinemets

**Affiliations:** ^1^Research Group in Plant Biology under Mediterranean Conditions, Department of Biology, Universitat de les Illes Balears, Carretera de Valldemossa km 7.5, 07122 Palma, Illes Balears, Spain; ^2^Institute of Agricultural and Environmental Sciences, Estonian University of Life Sciences, Kreutzwaldi 1, Tartu 51014, Estonia; ^3^Estonian Academy of Sciences, Kohtu 6, 10130 Tallinn, Estonia

**Keywords:** Activation energy, adaptation, carboxylation, meta-analysis, photosynthesis, temperature dependences.

## Abstract

The present study provides a synthesis of the *in vitro* and *in vivo* temperature responses of Rubisco Michaelis–Menten constants for CO_2_ (*K*_c_) and O_2_ (*K*_o_), specificity factor (*S*_c,o_) and maximum carboxylase turnover rate ($k_{cat}^{c}$) for 49 species from all the main photosynthetic kingdoms of life. Novel correction routines were developed for *in vitro* data to remove the effects of study-to-study differences in Rubisco assays. The compilation revealed differences in the energy of activation (∆*H*_a_) of Rubisco kinetics between higher plants and other photosynthetic groups, although photosynthetic bacteria and algae were under-represented and very few species have been investigated so far. Within plants, the variation in Rubisco temperature responses was related to species’ climate and photosynthetic mechanism, with differences in ∆*H*_a_ for $k_{cat}^{c}$ among C_3_ plants from cool and warm environments, and in ∆*H*_a_ for $k_{cat}^{c}$ and *K*_c_ among C_3_ and C_4_ plants. A negative correlation was observed among ∆*H*_a_ for *S*_c/o_ and species’ growth temperature for all data pooled, supporting the convergent adjustment of the temperature sensitivity of Rubisco kinetics to species’ thermal history. Simulations of the influence of varying temperature dependences of Rubisco kinetics on Rubisco-limited photosynthesis suggested improved photosynthetic performance of C_3_ plants from cool habitats at lower temperatures, and C_3_ plants from warm habitats at higher temperatures, especially at higher CO_2_ concentration. Thus, variation in Rubisco kinetics for different groups of photosynthetic organisms might need consideration to improve prediction of photosynthesis in future climates. Comparisons between *in vitro* and *in vivo* data revealed common trends, but also highlighted a large variability among both types of Rubisco kinetics currently used to simulate photosynthesis, emphasizing the need for more experimental work to fill in the gaps in Rubisco datasets and improve scaling from enzyme kinetics to realized photosynthesis.

## Highlight

A synthesis of existing data reveals differences in the temperature responses of Rubisco kinetics among higher plants, with important consequences for photosynthesis modeling.

## Introduction

According to the Farquhar, von Caemmerer and Berry (1980) model of C_3_ photosynthesis (FvCB model; Farquhar *et al.*, 1980; von Caemmerer, 2000), under physiologically relevant conditions, CO_2_ fixation rates are limited by the carboxylation of ribulose-1,5-bisphosphate (RuBP). RuBP carboxylation in turn is either limited by the regeneration of RuBP (typically by the rate of photosynthetic electron transport) or by the activity of the carboxylating enzyme, RuBP carboxylase/oxygenase (Rubisco). The limitations imposed by Rubisco result from its notorious catalytic inefficiencies, including slow catalysis and imperfect discrimination between CO_2_ and O_2_ (Whitney *et al.*, 2011). Due to these inefficiencies, plants need to accumulate high amounts of Rubisco, and lose significant amounts of previously fixed CO_2_ and NH_3_ in the process of photorespiration (Keys, 1986). Indeed, a slow rate of catalysis and competitive inhibition by O_2_ not only limit the rate of CO_2_ fixation, but also compromise the capacity of photosynthetic organisms for optimal use of water and nitrogen, the key limiting resources. Not surprisingly, Rubisco has been listed among the most obvious targets to improve the photosynthetic capacity of crops (Long *et al.*, 2006; Galmés *et al.*, 2014*a*). Theoretical estimations indicate that reducing these Rubisco inefficiencies could deliver increases in net photosynthesis of up to 60% in the mid-term (Murchie *et al.*, 2008; Zhu *et al.*, 2010).

The quantitative impacts of Rubisco inefficiencies depend on the environmental conditions during catalysis. For instance, under drought stress conditions, RuBP oxygenation is favored over carboxylation because of the lower concentration of CO_2_ ([CO_2_]) at the active sites of Rubisco due to reduced rate of CO_2_ diffusion through stomata and leaf mesophyll (Chaves *et al.*, 2009; Cornic and Massacci, 2004; Flexas *et al.*, 2004; Niinemets and Keenan, 2014). Furthermore, at any given [CO_2_], Rubisco catalysis is also strongly affected by temperature. In particular, the maximum carboxylase turnover rate ($k_{\text{cat}}^{\text{c}}$) of Rubisco increases up to 50–55 ºC or even higher for some organisms from extreme environments (Galmés *et al.*, 2015 for a review), while the Rubisco specificity factor (*S*_c/o_) decreases and the Michaelis–Menten constants for CO_2_ (*K*_c_) and O_2_ (*K*_o_) increase (Bernacchi *et al.*, 2001). Although the basic patterns of temperature-dependent variation in key Rubisco kinetic characteristics are well known and measured in a number of studies, there is surprisingly limited comparative information of the variability of temperature responses of Rubisco across different photosynthetic groups that evolved at different periods of time, as well as among photosynthetic organisms adapted to different environmental conditions.

Considerable variation exists in the catalytic properties of Rubisco among distant phylogenetic groups with different Rubisco types (Jordan and Ogren, 1981; Keys, 1986; Raven, 2000; Savir *et al.*, 2010; Whitney *et al.*, 2011), but also within closely related taxa (Galmés *et al.*, 2005, 2014*b*, *c*; Kubien *et al.*, 2008; Ishikawa *et al.*, 2009). Several lines of evidence suggest that the most likely factor shaping the specialization in Rubisco kinetics among higher plants is the availability of CO_2_ at the active sites of the enzyme in the chloroplastic stroma (Delgado *et al.*, 1995; Raven, 2000; Young *et al.*, 2012; Galmés *et al.*, 2014b, c). Importantly, optimization of Rubisco kinetic traits to the prevailing [CO_2_] has inevitably to deal with the trade-off between Rubisco affinity for CO_2_ and enzyme turnover rate (Badger and Andrews, 1987; Tcherkez *et al.*, 2006; Savir *et al.*, 2010). Thus, evolution in Rubisco catalytic properties in species with the C_4_ carbon concentration mechanism has led to increased $k_{\text{cat}}^{\text{c}}$ and *K*_c_ (Ghannoum *et al.*, 2005; Kubien *et al.*, 2008). In contrast, C_3_ species with lower [CO_2_] at the carboxylation site, especially species from dry, warm and saline habitats, are characterized by lower *K*_c_ and $k_{\text{cat}}^{\text{c}}$ (Galmés *et al.*, 2005, 2014*c*).

Similar to the [CO_2_]-driven evolution of Rubisco, other evidence suggests that the evolution of the enzyme’s catalytic traits has also been driven by the prevailing growth temperature (Sage, 2002; Galmés *et al.*, 2005; Tcherkez *et al.*, 2006; Yamori *et al.*, 2006; Cavanagh and Kubien, 2013). A broad compilation of the temperature dependences of $k_{\text{cat}}^{\text{c}}$ confirmed the existence of a notable natural variation in Rubisco thermal tolerance (Galmés *et al.*, 2015). More importantly, within the land plants, the energy of activation of $k_{\text{cat}}^{\text{c}}$ was positively correlated with the species’ thermal environment. A recent study further provided evidence that the evolutionary adjustment in the temperature sensitivity of Rubisco kinetic properties differed between C_3_ and C_4_ species of *Flaveria* (Perdomo *et al.*, 2015).

From a practical perspective, the accuracy of the FvCB model in simulating temperature responses of leaf photosynthesis for any given species requires information on the species-specific temperature dependences of the Rubisco catalytic constants, in particular, *K*_c_, *K*_o_, *S*_c/o_ (or the photosynthetic CO_2_ compensation point in the absence of mitochondrial respiration, Γ*) and $k_{\text{cat}}^{\text{c}}$ (von Caemmerer, 2000; Bernacchi *et al.*, 2001; Walker *et al.*, 2013). Indeed, recent modeling indicates that the temperature dependence of Rubisco kinetics dictates the optimum temperature for the photosynthetic rate (Galmés *et al.*, 2014*a*). So far, application of the FvCB model to leaf photosynthesis has used three main datasets of temperature dependences of Rubisco kinetics: Badger and Collatz (1977) for *Atriplex glabriuscula* (*in vitro* measurements, used also in the original FvCB model), Jordan and Ogren (1984) for *Spinacia oleracea* (*in vitro* measurements) and Bernacchi *et al.* (2001) for *Nicotiana tabacum* (determined from *in vivo* leaf gas-exchange measurements in transgenic lines with reduced Rubisco content where photosynthesis was assumed to be limited by Rubisco under all [CO_2_] and leaf temperature combinations). These three datasets are widely used in modeling photosynthesis of species, plant stands, landscapes and biomes, whereas the use of any one of the three datasets mainly reflects the historical roots of the given modeling community (e.g. Niinemets *et al.*, 2009a, b; Keenan *et al.*, 2010; Galmés *et al.*, 2011*a*; Bermúdez *et al.*, 2012; Bernacchi *et al.*, 2013; Bagley *et al.*, 2015; see also Niinemets *et al.*, 2015 for an analysis of the frequency of use of different Rubisco datasets across studies). Implicit in the use of a single species’ temperature response of Rubisco kinetics is that the variability among these responses is small across species spanning biomes with extensive variations in temperature and water availability. However, already comparisons of the *in vitro A. glabriuscula* and *S. oleracea* data and *N. tabacum in vivo* data have indicated that the variability is profound (Bernacchi *et al.*, 2001). More recently, Walker *et al.* (2013) compared *in vivo* temperature dependences of Rubisco catalytic constants between *Arabidopsis thaliana* and *N. tabacum* and demonstrated that species-dependent differences in Rubisco kinetics do alter simulations of leaf photosynthesis.

Overall, the need for accurate estimations of the temperature dependences of Rubisco kinetic parameters has become apparent as mathematical modelers try to predict the impact of increasing global temperatures on plant productivity (Sage and Kubien, 2007; Gornall *et al.*, 2010). As the natural variation in temperature responses of $k_{\text{cat}}^{\text{c}}$ has been analysed in a recent compilation (see above, Galmés *et al.*, 2015), temperature responses of $k_{\text{cat}}^{\text{c}}$ can be included in models separately for different species groups from warm and cold habitats, but no such synthetic analysis exists for temperature responses of other key Rubisco characteristics, *K*_c_, *K*_o_ and *S*_c/o_. Construction of such integrated datasets has been difficult due to limited *in vivo* data and multiple complications with *in vitro* measurements. Among such complications for *in vitro* studies are study-to-study differences in the assay medium composition and in the values of physico-chemical characteristics used in the estimation of the concentrations of CO_2_ and O_2_ in the assay medium (e.g. Yokota and Kitaoka, 1985 for highlighting the problem). The present work aims to fill this gap and to provide a comprehensive analysis of the available temperature responses of Rubisco kinetics. The specific objectives of our analysis were: (i) to compile all available temperature responses of Rubisco, and to normalize the temperature parameters of different species to standard conditions for comparative purposes, (ii) to examine differences in the temperature parameters for the Rubisco kinetics from different species, (iii) to determine whether these differences are related to the phylogeny and/or the ecology of the species, (iv) to compare the temperature parameters of Rubisco kinetics derived from *in vivo* and *in vitro* measurements, and (v) to quantify the impact of these differences on model estimates of Rubisco-limited photosynthesis.

## Methods

Data on the *in vitro* and *in vivo* Rubisco kinetic parameters, specificity factor (*S*_c/o_) and Michaelis–Menten constants for CO_2_ (*K*_c_) and O_2_ (*K*_o_) at varying temperature were compiled from peer-reviewed literature identified by Thompson-Reuters ISI Web of Science (Philadelphia, PA, USA).

### *In vitro* data compilation

The *in vitro* database consisted of the following information: article bibliographic data, species name, cultivar name for agricultural plants and strain name for bacteria (where reported), measurement temperature (*T*) and pH, the ionic composition of the assay buffer, headspace gas composition and, wherever available, the ionic strength of the assay solution and the acidity constant of dissolved CO_2_ ($\text{p}K_{{\text{a,CO}}_{2}}$) used to estimate the CO_2_ concentration in solution ($C_{{\text{CO}}_{2}\text{,liq}}$) from added bicarbonate concentration at given solution pH. Wherever relevant data were missing, article authors were contacted and obtained information was included in the database (see the Acknowledgements section).

The information about the assay buffer composition was needed to correct for study-to-study differences in the solution CO_2_ and O_2_ concentrations. In particular, the key issue with *in vitro* data is that the solubility of gases and the equilibrium coefficients of bicarbonate, which is commonly used as the source for CO_2_, depend on solution temperature and composition (Yokota and Kitaoka, 1985). While early studies have often used the O_2_, CO_2_ and bicarbonate equilibrium characteristics for pure water, it was later realized that $\text{p}K_{{\text{a,CO}}_{2}}$ depends on solution ionic strength (Yokota and Kitaoka, 1985) with major implications for estimates of *K*_c_. However, solution composition also affects the solubility of O_2_ and thereby the estimation of *K*_o_, and both differences in bicarbonate equilibrium and O_2_ solubility affect estimation of *S*_c/o_. Furthermore, equilibrium constants are needed to convert between gas- and liquid-phase equivalent values of *K*_c_, *K*_o_ and *S*_c/o_. This means that major differences among the estimates of these characteristics across studies can simply result from differences in the equilibrium constants used. Although the general importance of physico-chemical characteristics in Rubisco assays is well understood by the Rubisco research community, studies continue using different estimates of physico-chemical characteristics. In this study, the information on assay buffer characteristics was employed to correct for differences in Rubisco characteristics resulting from varying equilibrium coefficients used.

Across all analysed literature reporting *in vitro* data, the temperature response of *S*_c/o_ was obtained from 12 different studies: Badger and Collatz (1977), Jordan and Ogren (1984), Lehnherr *et al.* (1985), Uemura *et al.* (1997), Zhu *et al.* (1998), Galmés *et al.* (2005), Haslam *et al.* (2005), Yamori *et al.* (2006), Gubernator *et al.* (2008), Boyd *et al.* (2015), Perdomo *et al.* (2015) and Hermida-Carrera *et al.* (2016). These studies provided estimates for 38 species (*n*=1 for Proteobacteria and Rhodophyta; *n*=2 for Cyanobacteria; *n*=4 for Bacillariophyta; *n*=30 for Spermatophyta).

The temperature response of *in vitro K*_c_ was obtained from the following studies: Laing *et al.* (1974), Badger and Collatz (1977), Badger (1980), Jordan and Ogren (1984), Lehnherr *et al.* (1985), Castrillo (1995), Wei *et al.* (1994), Boyd *et al.* (2015), Perdomo *et al.* (2015), Young *et al.* (2015) and Hermida-Carrera *et al.* (2016) providing information for 21 species (Cyanobacteria, *n*=1; Bacillariophyta, *n*=2; Spermatophyta, *n*=18). Finally, data on the temperature response of *in vitro K*_o_ for five species (all Spermatophyta) were obtained from Laing *et al.* (1974), Badger and Collatz (1977), Jordan and Ogren (1984), Lehnherr *et al.* (1985) and Boyd *et al.* (2015).

The specific temperatures at which the Rubisco kinetic parameters were measured differed among the original studies, but compiled studies reported measurements for at least three different temperatures, except the data from Jordan and Ogren (1984) for *Rhodospirillum rubrum*, and Young *et al.* (2015) for *Thalassiosira weissflogii* and *Fragilariopsis cylindrus*, with two assayed temperatures.

Initially, four additional datasets were incorporated in the database, but were ultimately not used in the analyses due to following reasons. In the case of *Triticum aestivum*, the data of Hall and Keys (1983) for the temperature response of *S*_c/o_ and Mächler and Nösberger (1980) for the temperature response of *K*_c_ presented a large scatter. Analogously, *K*_c_ temperature response for *Agropyron smithii* in Monson *et al.* (1982) had a large scatter and evidence of non-monotonic temperature response (*r* of only 0.83 for the linear regression between measured and predicted values compared with *r*>0.95 for all other *in vitro K*_c_ data) and was therefore not included in comparison of *K*_c_ temperature responses. However, it was used in the comparison between *in vitro* and *in vivo* data to highlight the potential uncertainties among different types of data. The *S*_c/o_ for *Thermococcus kodakariensis* of Ezaki *et al.* (1999) increased with increasing the measurement temperature contrary to all other data, and these data were therefore deemed unreliable.

The temperature responses of $k_{\text{cat}}^{\text{c}}$ from Galmés *et al.* (2015) for 49 species (Archaea, *n*=1; Cyanobacteria, *n*=3; Proteobacteria, *n*=4; Rhodophyta, *n*=1; Chlorophyta, *n*=4; Spermatophyta, *n*=36) were also included for an integrated analysis of the relationships between the temperature dependence of the different kinetic parameters of Rubisco.

### Correction of *in vitro* data for differences in the equilibrium coefficients used

In the case of *K*_c_, the buffer composition-corrected value (*K*_c,c_) and measured (*K*_c,m_) value depend on the solution pH and used (p*K*_a,u_) and buffer composition-corrected (p*K*_a,c_) acidity constants of dissolved CO_2_. For the typical pH ranges used in Rubisco assays, the undissociated carbonic acid concentration is negligible (<10^−10^ M), and $\text{p}K_{{\text{a,CO}}_{2}}$ = −log([H^+^][HCO_3_^−^]/$C_{{\text{CO}}_{2}\text{,liq}}$), where [H^+^] is the hydrogen ion concentration and [HCO_3_^−^] the bicarbonate concentration. Thus, *K*_c,c_ is given as:

$$K_{\text{c,c}}=K_{\text{c,m}}\frac{\left(1-\frac{10^{\text{pH}-\text{p}K_{\text{a,c}}}}{1+10^{\text{pH}-\text{p}K_{\text{a,c}}}}\right)}{\left(1-\frac{10^{\text{pH}-\text{p}K_{\text{a,u}}}}{1+10^{\text{pH}-\text{p}K_{\text{a,u}}}}\right)}$$ (1)

Yokota and Kitaoka (1985) have proposed an equation to estimate p*K*_a,c_ values based on the solution ionic strength and temperature. However, their equation was based on only one study (Harned and Bonner, 1945), and included only three parameters such that it was accurate only over the temperature range of 10–35 °C (Yokota and Kitaoka, 1985). Because *K*_c,c_ depends highly non-linearly on p*K*_a,c_ (Eq. 1), we considered it essential to improve estimation of p*K*_a,c_. Thus, we conducted a meta-analysis of published p*K*_a,c_ values reported for different solution temperatures (0–50 °C) and ionic strengths (0–1.042M). Altogether, 105 estimates of p*K*_a,c_ were obtained (Shedlovsky and MacInnes, 1935; Harned and Davis, 1943; Harned and Bonner, 1945; Pocker and Bjorkquist, 1977; Schumacher and Smucker, 1983; Yokota and Kitaoka, 1985). To convert the molal concentrations reported in some studies, including Harned and Bonner (1945), to the corresponding molar concentrations, water density at different solute concentrations and temperatures was estimated using a polynomial equation based on data of Weast (1974).

Consistent with the Debye–Hückel theory of non-ideality of solutions, and as common in analytical chemistry studies fitting p*K*_a_ values of different buffer substances (e.g. Bates *et al.*, 1973; Roy *et al.*, 1998; Roy *et al.*, 2009), we have used a series of log and polynomial terms to describe the dependence of p*K*_a,c_ on absolute temperature (*T*_k_, K) and solution ionic strength (*I*_s_, M) as:

$$\begin{array}{ll} \text{p}K_{\text{a,c}} & =\frac{a_{1}}{T_{\text{k}}}+a_{2}\log\left(T_{\text{k}}\right)+a_{3}T_{\text{k}}+a_{4}+s_{1}I_{\text{s}}\\ & +s_{2}\sqrt{I_{\text{s}}}+s_{\text{3}}I_{\text{s}}{}^{2}+s_{4}I_{\text{s}}{}^{3}, \end{array}$$ (2)

where the values of the empirical coefficients are: *a*_1_=16400, *a*_2_=211.56, *a*_3_=−0.1291, *a*_4_=−533.63, *s*_1_=0.3252, *s*_2_=0.3830, *s*_3_=−0.2692, *s*_4_=−0.8503. Equation 2 provided an excellent fit to the data (*r*^2^=0.9985, mean squared error of 4.8×10^−5^), i.e. a considerable improvement compared with the equation of Yokota and Kitaoka (1985) (*r*^2^=0.981 for the *T*_k_ and *I*_s_ range over which the equation was valid).

The ionic strength of the assay buffer was calculated considering all the ionic species in the solution. For weak acids and bases, including buffer substances, their p*K*_a_ values were used to estimate the concentration of the ionic species in the solution. Again, multiple regression equations similar to Eq. 2 were developed for individual compounds to consider p*K*_a_ dependences on *T*_k_ and *I*_s_ based on published data (e.g. Bates *et al.*, 1973; Feng *et al.*, 1989; Roy *et al.*, 2006, 2009, 2011). However, in the case of zwitterionic buffers, e.g. for HEPES (Vega and Bates, 1976; Feng *et al.*, 1989; Roy *et al.*, 2009) and Bicine (Datta *et al.*, 1964; Azab *et al.*, 1994; Roy *et al.*, 2006), the effect of *I*_s_ was not always important (but see Bates and Hetzer, 1961; Durst and Staples, 1972; Bates and Robinson, 1973; Ramette *et al.*, 1977 for the *I*_s_ dependence of Tris). Given the p*K*_a_ dependence on *I*_s_ and the *I*_s_ dependence on p*K*_a_, *I*_s_ and p*K*_a_ for the given buffer solution were ultimately calculated iteratively.

In the case of *K*_o_ estimations, the gas-phase oxygen concentration $(C_{{\text{O}}_{2}\text{,gas}}$, mol mol^−1^) was typically varied to achieve variation in the liquid-phase oxygen concentration $(C_{{\text{CO}}_{2}\text{,liq}}$, mol m^−3^). Thus, the key issue is how $C_{{\text{O}}_{2}\text{,gas}}$ has been converted in $C_{{\text{CO}}_{2}\text{,liq}}$. The concentrations in different phases are related through the Henry’s law constant ($H_{{\text{pc,O}}_{2}}$, Pa m^3^ mol^−1^) as:

$$H_{\text{pc},{\text{O}}_{2}}=\frac{C_{{\text{O}}_{2}\text{,gas}}}{PC_{{\text{O}}_{2}\text{,liq}}},$$ (3)

where *P* (Pa) is the air pressure. We note that the word ‘constant’ is misleading, because *H*_pc_ for the given compound depends on temperature and other solutes that can affect the solubility of the compound of interest (Sander, 2001; Staudinger and Roberts, 2001; Copolovici and Niinemets, 2007). Dependence of *H*_pc_ on the presence of solutes has been largely ignored by the Rubisco community. Here, we use different subscripts to clearly denote these effects.

Several different conversion factors taken from physical chemical reference sources had been used across the studies (data not shown), but all of these factors were based on pure water. However, oxygen solubility is importantly driven by the solute concentrations (Tromans, 2000; Gnaiger, 2001; Millero and Huang, 2003). The overall solubility in complex media such as biological assay buffers is difficult to predict due to partly non-additive effects of different solutes (e.g. Gros *et al.*, 1999). Thus, we have employed a simplified approach, and linked *H*_pc_ to total concentration of ions in solution. First, the value of *H*_pc_ for pure water, *H*_pc,0_, was described in dependence on temperature using a polynomial equation in the form:

$$H_{\text{pc},0}=c_{1}T_{\text{k}}{}^{3}+c_{2}T_{\text{k}}{}^{2}+c_{3}T_{\text{k}}{}^{3}+c_{4},$$ (4)

where the empirical coefficients have values *c*_1_=0.051816, *c*_2_=−42.437, *c*_3_=12977.3, *c*_4_=−1388072.1, which were derived from Millero and Huang (2003) and Millero *et al.* (2003). The value of *H*_pc_ corresponding to different solute concentrations, *C*_s_ (*H*_pc,s_) was further described as:

$$H_{\text{pc,s}}=\frac{H_{\text{pc,0}}}{\exp\left[\left(d_{1}+\frac{d_{2}}{T_{\text{k}}}+\frac{d_{3}}{\ln T_{\text{k}}}\right)C_{\text{s}}+\left(d_{4}+\frac{d_{5}}{T_{\text{k}}}+d_{6}\ln T_{\text{k}}\right)C_{\text{s}}{}^{2}\right]},$$ (5)

where *d*_1_–*d*_6_ are empirical coefficients that we initially derived for different electrolytes. As the differences among electrolytes were small and due to difficulties with the non-additivity mentioned above, in this analysis, we used the empirical coefficients *d*_1_=1.4565, *d*_2_=−178.90, *d*_3_=−6.0556, *d*_4_=−0.7818, *d*_5_=54.240, *d*_6_=0.10796 derived for KCl (*r*^2^=0.9983 for the complete fit including both the temperature effects on $H_{{\text{pc,0,CO}}_{2}}$ described by Eq. 4 and the denominator) based on the data of Millero and colleagues (Millero *et al.*, 2002, 2003; Millero and Huang, 2003). The implication of Eq. 5 is that the O_2_ solubility in ionic media is less, *ca* 3% at 0 °C and 1.5% at 50 °C, than in pure water (the salting-out effect, Table 1). Ultimately, the liquid-phase *K*_o_ values reported, *K*_o,m_, were corrected for solute effects as:

**Table 1.** Henry’s law constants (Pa m^3^ mol^−1^) for conversion of Rubisco kinetic characteristics among gas- and liquid-phase equivalent values (Eqs 8–10)

Gas	Medium	Temperature (ºC)			
		15	25	35	45
CO_2_	Pure water	2186	2982	3867	4777
CO_2_	Chloroplast	2230	3041	3944	4873
O_2_	Pure water	67 510	82 080	97 430	113 870
O_2_	Chloroplast	69 260	83 950	99 370	115 840

Henry’s law constant (*H*_pc_) is the gas–liquid phase equilibrium partition coefficient and is given as the ratio of the gas partial pressure (Pa) and corresponding liquid-phase concentration (mol m^−3^, Eq. 3). Because the gas solubility depends on the presence of other solutes (salting-out effect), *H*_pc_ typically increases with increasing solute concentration. Equation 4 was used to estimate values of *H*_pc_ at different temperatures for pure water, and Eq. 5 for chloroplastic water. In the latter calculation, the dominant solute was assumed to be KCl and the solute concentration was taken as 0.11M. Derivation of Eqs 4 and 5 with supporting references and review of chloroplast solute concentrations is provided in the Methods.

$$K_{\text{o,c}}=K_{\text{o,m}}\frac{H_{\text{pc,u}}{}_{,O_{2}}}{H_{\text{pc,s}}{}_{,O_{2}}},$$ (6)

where *H*_pc,u_ is the value of the Henry’s law constant used at the given temperature in the original studies.

Estimates of the specificity factor, *S*_c/o_, depend on both differences in p*K*_a_ values used for the acidity constant of dissolved CO_2_ (p*K*_a,u_) and Henry’s law constant for O_2_. Thus, the *S*_c/o_ measurements, *S*_c/o,m_, were converted to a common set of equilibrium coefficients as:

$$S_{\text{c/o,c}}=S_{\text{c/o,m}}\frac{\left(1-\frac{10^{\text{pH}-\text{p}K_{\text{a,u}}}}{1+10^{\text{pH}-\text{p}K_{\text{a,u}}}}\right)H_{\text{pc,u}}{}_{,O_{2}}}{\left(1-\frac{10^{\text{pH}-\text{p}K_{\text{a,c}}}}{1+10^{\text{pH}-\text{p}K_{\text{a,c}}}}\right)H_{\text{pc,s}}{}_{,O_{2}}}.$$ (7)

### *In vivo* database

In the case of *in vivo* estimates (only available for Spermatophyta), the database included the following: article bibliographic data, species name, cultivar name for agricultural plants, measurement temperature (*T*) and details of the measurement methods used (e.g. gas exchange combined with chlorophyll fluorescence, gas exchange and carbon isotopic discrimination, gas exchange and ^14^CO_2_ uptake, indicating whether the leaf mesophyll conductance and mitochondrial respiration were considered when deriving Rubisco kinetic parameters). Typically, values of *K*_c_, *K*_o_ and/or *S*_c/o_ were reported for the gas phase (*K*_c,g_, *K*_o,g_, and *S*_c/o,g_), but when available, liquid-phase equivalent values of these characteristics and corresponding values of Henry’s law constants used were also included in the database. The gas-phase estimates of Rubisco characteristics estimated in *in vivo* studies themselves do not require any standardization, but as the Rubisco reaction takes place in the liquid phase, corresponding liquid-phase estimates are needed to compare Rubisco kinetics among species. The gas-phase estimates of Rubisco kinetic characteristics were converted to liquid-phase equivalent values (*K*_c,liq_, *K*_o,liq_, *S*_c/o,liq_) using the following equations:

$$K_{\text{c,liq}}=\frac{K_{\text{c,g}}}{H_{{\text{pc,s, CO}}_{2}}}P$$ (8)

$$K_{\text{o,liq}}=\frac{K_{\text{o,g}}}{H_{{\text{pc,s,O}}_{2}}}P$$ (9)

$$S_{\text{c,o,liq}}=\frac{S_{\text{c/o,g}}H_{{\text{pc,s,CO}}_{2}}}{H_{{\text{pc,s,O}}_{2}}},$$ (10)

where the Henry’s law constant for O_2_, $H_{{\text{pc,s,O}}_{2}}$, is given by Eq. 5 and that for CO_2_, *H*_pc,s,CO2_, was derived analogously (Table 1 for estimates of Henry’s law constant at different temperatures). For pure water, the temperature dependence of $H_{{\text{pc,0,CO}}_{2}}$ was derived analogously to that for O_2_ (Eq. 4) using an extensive set of values from published studies (van Slyke *et al.*, 1928; Markham and Kobe, 1940; Harned and Davis, 1943; Umbreit *et al.*, 1972; Rischbieter *et al.*, 1996) to estimate the empirical coefficients as *c*_1_=−0.01081, *c*_2_=10.1188, *c*_3_=− 3065.93 and *c*_4_=304097.1 (*r*^2^=0.9994). These reference sources were further employed to fit Eq. 5 to solute concentrations, and values of *d*_1_=−2.8858, *d*_2_=−173.31, *d*_3_=18.718, *d*_4_=0.41177, *d*_5_=−32.719, *d*_6_=−0.050167 were derived for KCl (*r*^2^=0.9993 for the complete fit including both the temperature effects on $H_{{\text{pc,0,CO}}_{2}}$ as described by Eq. 4 and the denominator). KCl was used as K is the dominant solute in plant cells (Gupta and Berkowitz, 1988; Schröppel-Meier and Kaiser, 1988), but analogous fits using other electrolytes such as NaCl were similar (data not shown). Given that ionic concentrations in chloroplasts of non-stressed leaves are on the order of 0.09–0.15M in non-dissociated salt equivalents used in developing Eq. 5 (Gupta and Berkowitz, 1988; Schröppel-Meier and Kaiser, 1988), we have taken *C*_s_ as 0.11M in this analysis. For comparison, an equivalent average value of 0.08M was estimated across the *in vitro* studies (assuming a non-dissociated salt consisting of two monovalent ions). When *in vivo* studies reported liquid-phase equivalent values of Rubisco kinetic characteristics, they were again converted to a common set of equilibrium constants. In the case of *K*_c_ and *K*_o_, Eq. 6 was used with corresponding Henry’s law constant values for CO_2_ and O_2_, while the values of *S*_c/o_ were standardized as:

$$S_{\text{c/o,c}}=S_{\text{c/o,m}}\frac{H_{\text{pc,u}}{}_{,{\text{O}}_{2}}H_{\text{pc,s}}{}_{{\text{CO}}_{2}}}{H_{\text{pc,u}}{}_{,{\text{CO}}_{2}}H_{\text{pc,s}}{}_{,{\text{O}}_{2}}}.$$ (11)

*In vivo* data on the temperature response of *S*_c/o_ were obtained from five different studies: Brooks and Farquhar (1985), Ghashghaie and Cornic (1994), Bernacchi *et al.* (2001), Viil *et al.* (2012) and Walker *et al.* (2013) providing information for six species. The temperature responses of *in vivo K*_c_ were obtained from the following studies: Monson *et al.* (1982), Harley *et al.* (1985), Bernacchi *et al.* (2001) and Walker *et al.* (2013) yielding data for four species. The temperature responses of *in vivo K*_o_ for three species were extracted from Harley *et al.* (1985), Bernacchi *et al.* (2001) and Walker *et al.* (2013). Across all these studies, mesophyll conductance (*g*_m_) had been considered, and accordingly Rubisco kinetics based on chloroplastic CO_2_ concentration had been derived, only in the study of Walker *et al.* (2013). In all other studies, Rubisco kinetics had been derived based on intercellular CO_2_ concentration. Although Bernacchi *et al.* (2002) have reported the temperature kinetics of *g*_m_, the Rubisco temperature characteristics of Bernacchi *et al.* (2001) cannot be readily converted to chloroplastic CO_2_ concentration based estimates using these independent measurements of *g*_m_. This is because at any given value of *g*_m_, the CO_2_ drawdown between the intercellular airspace and chloroplasts can vary due to differences in foliage anatomical characteristics and leaf photosynthetic capacity (Niinemets *et al.*, 2009*a*; Tomás *et al.*, 2013).

### Species’ phylogenetic and ecological characteristics grouping

Species were grouped into the main phylogenetic groups Archaea, Proteobacteria, Cyanobacteria, Rhodophyta, Chlorophyta, Bacillariophyta and Spermatophyta. The average optimum growth temperature (*T*_growth_) for each species was obtained from the literature or assigned according to the species’ climatic range as in Galmés *et al.* (2015). Spermatophytes were further classified according to their photosynthetic mechanism and *T*_growth_ as C_3_ plants from warm environments (*T*_growth_≥25 ºC), C_3_ plants from cool environments (*T*_growth_<25 ºC) and C_4_ plants. The arbitrary threshold of 25 ºC was based on analogous studies (Sage, 2002; Galmés *et al.*, 2015).

### Fitting the temperature responses

The temperature response curves of the kinetic parameters obtained from the original data were fitted for each individual temperature response dataset by an Arrhenius-type temperature response function:

$$f(T)=e^{c-\Delta H_{\text{a}}/RT}$$ (12)

where *c* is the scaling constant for the parameter, ∆*H*_a_ (J mol^−1^) is the activation energy, *T* (K) is the temperature and *R* is the universal gas constant (8.314 J mol^−1^ K^−1^). Equation 12 was fitted to the data by iteratively minimizing the sum of squares between the measured and predicted values of each kinetic parameter using the Microsoft Excel Solver function.

The temperature response curves of the kinetic parameters were also fitted by second- and third-order polynomial equations in the form of:

$$f(T)=a+bT+cT^{2}$$ (13)

$$f(T)=a+bT+cT^{2}+dT^{3}$$ (14)

As the actual measurement temperatures differed across studies, the Arrhenius-type and polynomial equations (Eqs 12–14) were further used to calculate the standardized values of each parameter at 5, 15, 25, 35 and 45 ºC for each species. These values were used to obtain the *Q*_10_ value over the temperature intervals of 5–15, 15–25, 25–35 and 35–45 ºC. We also reanalysed the $k_{\text{cat}}^{\text{c}}$ temperature data described in Galmés *et al.* (2015) to calculate the *Q*_10_ values.

To compare the different functions, the correlation coefficient (*r*) was calculated for linear regressions for predicted *vs*. measured values by SigmaPlot 11.0 (Systat Software, Inc., San Jose, CA, USA) as a measure of goodness of the fits.

### Simulation of temperature responses of Rubisco-limited photosynthesis

We used the photosynthesis model of Farquhar *et al.* (1980) to quantify the importance, in terms of Rubisco-limited CO_2_ gross assimilation rate (*A*_Rubisco_), of different thermal sensitivities of Rubisco kinetics from C_3_ plants from cool and warm environments. *A*_Rubisco_ only provides the potential estimate of photosynthesis rate supported by a given set of Rubisco characteristics under RuBP-saturated conditions (Farquhar *et al.* 1980). The extent to which this potential is realized depends on the rate of RuBP regeneration, which in turn is driven by the actual light level, the capacity of RuBP regeneration (typically determined by the capacity for photosynthetic electron transport) and the temperature dependence of RuBP regeneration (Farquhar *et al.* 1980; Galmés *et al.* 2014*a*). Although this complicates interpretation of differences in *A*_Rubisco_ temperature responses, we note that over the ambient temperature range of 15–40 ºC, Rubisco characteristically operates in RuBP-saturated conditions at higher light (photosynthetic quantum flux density greater than *ca* 400 μmol m^−2^ s^−1^) and lower CO_2_ concentrations (chloroplastic CO_2_ concentration, *C*_c_, less than *ca* 200 μmol mol^−1^) with the actual crossover between Rubisco-limited and RuBP-limited conditions depending on combinations of light, *C*_c_, temperature and temperature responses of *A*_Rubisco_ and RuBP regeneration (Farquhar *et al.* 1980; Galmés *et al.* 2014*a*). In these simulations, group-specific average temperature parameters for *S*_c/o_, *K*_c_ and $k_{\text{cat}}^{\text{c}}$ were used (Table 3), while the temperature dependence parameters of *K*_o_ were considered invariable among plant functional types. They were obtained as the average of the four reported *in vitro* values for C_3_ plants (*c*=9.9 and ∆*H*_a_=9.7 kJ mol^−1^, see Table 2 for single species data). A value of $k_{\text{cat}}^{\text{c}}$ of 2.5s^−1^ at 25 ºC and a leaf Rubisco content of 2g m^−2^ (equivalent to a concentration of 29 μmol catalytic sites m^−2^) were used for all plant functional types. The values of the deactivation energy (∆*H*_d_) and the entropy term (∆*S*) for $k_{\text{cat}}^{\text{c}}$ used for the different plant functional types were taken from Galmés *et al.* (2015): C_3_ plants from cool habitats, ∆*H*_d_=305 kJ mol^−1^, ∆*S*=929 J mol^−1^ K^−1^; C_3_ plants from warm habitats, ∆*H*_d_=220 kJ mol^−1^, ∆*S*=664 J mol^−1^ K^−1^; C_3_ average, ∆*H*_d_=258 kJ mol^−1^, ∆*S*=782 J mol^−1^ K^−1^.

**Table 2.** Temperature dependence parameters of *in vitro*-measured Rubisco catalytic constants

Group	Species	Reference	*T*_meas_ (ºC)	*c*	∆*H*_a_ (kJ mol^−1^)	*r*	$Q_{10}^{{}^{15\;–5}}$	$Q_{10}^{25–15}$	$Q_{10}^{{}^{35–25}}$	$Q_{10}^{{}^{45–35}}$	*T*_growth_ (ºC)
*S*_c/o_											
Proteobacteria	*Rhodospirillum rubrum*^*a*^	Jordan and Ogren (1984)^*a*^	2–25	−5.2	−18.8	n.d.	0.75	0.77			33
Cyanobacteria	*Thermosynechococcus elongatus* BP-1	Gubernator *et al.* (2008)	15–45	−13.0	−43.0	0.998	0.52	0.55	0.57	0.59	56
	*Synechococcus lividus*	Zhu *et al.* (1998)	15–35	−0.9	−11.7	0.999	0.84	0.85	0.86	0.87	45
Rhodophyta	*Galdieria partita* Tokara	Uemura *et al.* (1997)	15–45	−10.3	−38.7	0.991	0.56	0.58	0.60	0.62	45
Bacillariophyta	*Chaetoceros socialis*	Haslam *et al.* (2005)	10–25	−3.2	−18.7	0.975	0.76	0.77	0.78	0.80	10
	*Skeletonema costatum*	Haslam *et al.* (2005)	10–25	−7.1	−28.1	0.992	0.66	0.67	0.69	0.71	20
	*Thalassiosira antarctica*	Haslam *et al.* (2005)	10–25	−3.8	−20.3	0.963	0.74	0.75	0.77	0.78	−0.5
	*Thalassiosira hyalina*	Haslam *et al.* (2005)	10–25	−2.9	−18.1	0.991	0.76	0.78	0.79	0.80	2
Spermatophyta (C_3_ plants from cool habitats)	*Atriplex glabriuscula*	Badger and Collatz (1977)^*b*^	15–35	−0.1	−12.1	0.984	0.76	0.81	0.90	1.05	20
	*Avena sativa* cv. Forridena	Hermida-Carrera *et al.* (2016)^*c*^	15–35	−2.9	−18.4	0.998	0.76	0.77	0.79	0.80	20
	*Hordeum vulgare* ssp. *vulgare* cv. Morex	Hermida-Carrera *et al.* (2016)^*^c^*^	15–35	−2.5	−17.4	0.997	0.77	0.78	0.80	0.81	20
	*Lysimachia minoricensis*	Galmés et al. (2005)	15–35	−4.2	−21.4	0.989	0.73	0.74	0.76	0.77	20
	*Mentha aquatica*	Galmés et al. (2005)	15–35	−4.2	−21.4	0.996	0.73	0.74	0.76	0.77	20
	*Spinacea oleracea*	Uemura *et al.* (1997)	15–35	−3.5	−19.8	0.996	0.74	0.76	0.77	0.78	16
	*Spinacea oleracea*	Zhu *et al.* (1998)	15–35	−2.6	−17.6	0.999	0.77	0.78	0.79	0.81	16
	*Spinacea oleracea*	Jordan and Ogren (1984)	7–25	−3.8	−20.5	0.999	0.77	0.75	0.74	0.74	16
	*Spinacea oleracea* cv. Torai	Yamori *et al.* (2006)^*^d^*^	5–45	−1.4	−14.7	0.987	0.80	0.81	0.83	0.84	16
	*Spinacea oleracea*	Average		−3.3	−19.3		0.77	0.78	0.78	0.79	16
	*Triticum aestivum*	Haslam *et al.* (2005)	10–25	−3.7	−20.5	0.997	0.74	0.75	0.76	0.78	20
	*Triticum aestivum*	Hermida-Carrera *et al.* (2016)^*c*^	15–35	−3.4	−19.7	0.999	0.74	0.76	0.77	0.79	20
	*Triticum aestivum*	Average		−3.6	−20.0		0.74	0.75	0.77	0.78	20
	*Urtica atrovirens* ssp*. bianorii*	Galmés et al. (2005)	15–35	−4.1	−21.1	0.999	0.73	0.74	0.76	0.77	20
Spermatophyta (C_3_ plants from warm habitats)	*Beta maritima* ssp*. marcosii*	Galmés et al. (2005)	15–35	−4.4	−22.0	0.999	0.72	0.73	0.75	0.76	25
	*Beta maritima* ssp*. maritima*	Galmés et al. (2005)	15–35	−4.5	−22.4	0.994	0.71	0.73	0.75	0.76	25
	*Diplotaxis ibicensis*	Galmés et al. (2005)	15–35	−5.6	−24.9	0.998	0.69	0.71	0.72	0.74	25
	*Flaveria cronquistii*	Perdomo *et al.* (2015)	10–40	−3.5	−19.5	0.985	0.75	0.76	0.77	0.79	30
	*Flaveria pringlei*	Zhu *et al.* (1998)	15–35	−3.0	−18.9	0.999	0.75	0.77	0.78	0.79	30
	*Flaveria pringlei*	Perdomo *et al.* (2015)	10–40	−4.0	−20.8	0.991	0.73	0.75	0.76	0.77	30
	*Flaveria pringlei*	Average		−3.5	−19.8		0.7	0.8	0.8	0.8	30
	*Hypericum balearicum*	Galmés et al. (2005)	15–35	−4.7	−22.6	0.999	0.71	0.73	0.74	0.76	25
	*Kundmannia sicula*	Galmés et al. (2005)	15–35	−4.9	−23.2	0.996	0.71	0.72	0.74	0.75	25
	*Limonium gibertii*	Galmés et al. (2005)	15–35	−5.1	−24.1	0.999	0.70	0.71	0.73	0.74	25
	*Limonium magallufianum*	Galmés et al. (2005)	15–35	−5.2	−24.3	0.998	0.69	0.71	0.73	0.74	25
	*Pistacia lentiscus*	Galmés et al. (2005)	15–35	−4.4	−22.0	0.999	0.72	0.74	0.75	0.76	25
	*Rhamnus alaternus*	Galmés et al. (2005)	15–35	−4.7	−22.8	0.998	0.71	0.73	0.74	0.76	25
	*Rhamnus ludovici-salvatoris*	Galmés et al. (2005)	15–35	−5.2	−24.0	0.993	0.70	0.71	0.73	0.74	25
	*Trifolium repens* (native ecotype)	Lehnherr *et al.* (1985)	10–25	−2.8	−18.0	0.983	0.76	0.78	0.79	0.80	25
	*Urtica membranacea*	Galmés *et al.* (2005)	15–35	−3.7	−20.4	0.998	0.74	0.75	0.77	0.78	25
	*Flaveria angustifolia* (C_3_–C_4_)	Perdomo *et al.* (2015)	10–40	−4.3	−21.6	0.978	0.72	0.74	0.75	0.77	30
	*Flaveria floridana* (C_3_–C_4_)	Perdomo *et al.* (2015)	10–40	−3.8	−20.4	0.989	0.74	0.75	0.77	0.78	30
Spermatophyta (C_4_ plants)	*Amaranthus hybridus*	Jordan and Ogren (1984)	5–35	−7.0	−27.9	0.998	0.66	0.68	0.69	0.71	30
	*Flaveria bidentis*	Perdomo *et al.* (2015)	10–40	−3.8	−20.0	0.994	0.74	0.76	0.77	0.78	30
	*Flaveria trinervia*	Perdomo *et al.* (2015)	10–40	−4.3	−21.4	0.999	0.73	0.74	0.76	0.77	30
	*Saccharum officinarum*	Hermida-Carrera *et al.* (2016)^*c*^	15–35	−4.9	−23.0	0.997	0.71	0.72	0.74	0.75	30
	*Setaria viridis*	Boyd *et al.* (2015)	10–40	−4.6	−21.3	0.950	0.73	0.74	0.76	0.77	25
	*Zea mays* cv. Carella	Hermida-Carrera *et al.* (2016)^*c*^	15–35	−3.6	−20.1	0.999	0.74	0.75	0.77	0.78	30
*K*_c_											
Cyanobacteria	*Anabaena variabilis* M3	Badger (1980)	15–40	20.8	38.8	0.989	1.79	1.72	1.66	1.61	35
Bacillariophyta	*Fragilariopsis cylindrus*	Young *et al.* (2015)^*e*^	0–20	17.7	34.9	n.d.	1.69	1.63			5
	*Thalassiosira weissflogii*	Young *et al.* (2015)^*e*^	0–20	21.1	43.0	n.d.	1.91	1.83			22
Spermatophyta (C_3_ plants from cool habitats)	*Atriplex glabriuscula*	Badger and Collatz (1977)	5–35	15.9	32.4	0.987	1.63	1.57	1.53	1.49	20
	*Avena sativa* cv. Forridena	Hermida-Carrera *et al.* (2016)^*c*^	15–35	20.2	44.2	0.999	1.94	1.86	1.78	1.72	20
	*Espeletia schultzii*	Castrillo (1995)^*f*^	5–35	11.9	23.7	0.988	1.50	1.38	1.31	1.26	20
	*Hordeum vulgare* ssp. *vulgare* cv. Morex	Hermida-Carrera *et al.* (2016)^*c*^	15–35	16.2	34.6	0.999	1.68	1.62	1.57	1.53	20
	*Spinacia oleracea*	Jordan and Ogren (1984)	7–35	22.4	50.2	0.994	2.12	2.02	1.93	1.85	16
	*Triticum aestivum* cv. Cajeme	Hermida-Carrera *et al.* (2016)^*c*^	15–35	19.0	41.3	0.990	1.86	1.78	1.72	1.66	20
Spermatophyta (C_3_ plants from warm habitats)	*Flaveria cronquistii*	Perdomo *et al.* (2015)	10–40	22.9	51.8	0.996	2.18	2.07	1.97	1.89	30
	*Flaveria pringlei*	Perdomo *et al.* (2015)	10–40	17.7	38.6	0.983	1.78	1.72	1.66	1.60	30
	*Glycine max* cv. Wayne	Laing *et al.* (1974)	15–35	17.8	37.0	0.963	1.74	1.68	1.62	1.57	25
	*Oryza sativa indica*×*japonica* hybrid	Wei *et al.* (1994)	20–40	25.6	58.4	0.978	2.40	2.26	2.15	2.05	25
	*Trifolium repens* (native ecotype)	Lehnherr *et al.* (1985)	10–25	22.5	48.9	0.999	2.08	1.98	1.90	1.82	25
	*Flaveria angustifolia* (C_3_–C_4_)	Perdomo *et al.* (2015)	10–40	20.0	44.0	0.998	1.93	1.85	1.78	1.71	30
	*Flaveria floridana* (C_3_–C_4_)	Perdomo *et al.* (2015)	10–40	19.9	44.1	0.999	1.94	1.85	1.78	1.72	30
Spermatophyta (C_4_ plants)	*Flaveria bidentis*	Perdomo *et al.* (2015)	10–40	15.4	31.6	0.993	1.61	1.56	1.51	1.47	30
	*Flaveria trinervia*	Perdomo *et al.* (2015)	10–40	15.4	31.7	0.992	1.61	1.56	1.51	1.47	30
	*Saccharum officinarum*	Hermida-Carrera *et al.* (2016)^*c*^	15–35	17.8	35.8	0.998	1.71	1.65	1.60	1.55	30
	*Setaria viridis*	Boyd *et al.* (2015)	10–40	24.7	51.8	0.990	2.17	2.06	1.97	1.89	25
	*Zea mays* cv. Carella	Hermida-Carrera *et al.* (2016)^*c*^	15–35	12.6	22.9	0.971	1.41	1.38	1.35	1.32	30
*K*_o_											
Spermatophyta (C_3_ plants from cool habitats)	*Atriplex glabriuscula*	Badger and Collatz (1977)	15–35	19.7	34.6	0.996	1.68	1.62	1.57	1.53	20
	*Spinacia oleracea*	Jordan and Ogren (1984)^*g*^	7–35	6.2	0.0		1.00	1.00	1.00	1.00	16
Spermatophyta (C_3_ plants from warm habitats)	*Glycine max* cv. Wayne	Laing *et al.* (1974)	15–35	3.7	−5.5	0.989	0.92	0.93	0.93	0.94	25
	*Trifolium repens* (native ecotype)	Lehnherr *et al.* (1985)	10–25	10.1	9.3	0.927	1.15	1.14	1.13	1.12	25
Spermatophyta (C_4_ plants)	*Setaria viridis*	Boyd *et al.* (2015)	10–40	4.5	−4.0	0.738	0.94	0.95	0.95	0.95	25

Species were assigned to different phylogenetic groups. One phylogenetic group, Spermatophyta, was further divided into C_3_ and C_4_ species, and C_3_ species were further divided into warm- and cool-temperature species according to their optimum growth temperature (*T*_growth_). The two C_3_–C_4_ intermediate species *Flaveria angustifolia* and *F. floridana* were assigned to the group of C_3_ plants from warm habitats because they present C_3_-like Rubisco kinetics (Perdomo *et al.*, 2015). The optimum growth temperature (*T*_growth_) for each species was either obtained from literature or assigned according to their climate of origin. For *Spinacea oleracea*, *Triticum aestivum* and *F. pringlei*, individual reports’ values and average values for *S*_c/o_ of different reports are given. *c*, scaling constant; ∆*H*_a_, activation energy; *K*_c_, Michaelis–Menten constant for CO_2_; *K*_o_, Michaelis–Menten constant for O_2_; *r*, correlation coefficient for linear regressions between measured *vs*. predicted (Microsoft Excel Solver function) values of each kinetic parameter at the assayed temperatures; *Q*_10_, coefficient over the temperature intervals of 5–15 ºC ($Q_{10}^{{}^{15-5}}$), 15–25 ºC ($Q_{10}^{{}^{25-15}}$), 25–35 ºC ($Q_{10}^{{}^{35-25}}$) and 35–45 ºC ($Q_{10}^{{}^{45-35}}$); *S*_c/o_, Rubisco specificity factor for CO_2_/O_2_; *T*_meas_, range of measurement temperature.

n.d.: *r* was not determined because measurements consisted in only two assay temperatures.

^*a*^ Data from Jordan and Ogren (1984) for *Rhodospirillum rubrum* consisted of only two measurement temperatures (2 and 25 ºC) and, therefore, $Q_{10}^{{}^{35-25}}$ and $Q_{10}^{{}^{45-35}}$ were not calculated.

^*b*^ Due to poor convergence in the Excel Solver (low degree of explained variance), *c* and ∆*H*_a_ for this report were not considered in determining the group averages, and *Q*_10_ values were obtained from second-order polynomial fits. Due to high scatter at higher temperature, values of $Q_{10}^{{}^{35-25}}$ and $Q_{10}^{{}^{45-35}}$ from polynomial fits were also unreliable and were therefore not considered for group averages.

^*c*^ Data from Hermida-Carrera (2016) consisted of measurements at three temperatures (15, 25 and 35 ºC), and the assays were performed following the procedures described in Galmés *et al.* (2014*b*).

^*d*^ Low adjustment of the Excel Solver; *c* and ∆*H*_a_ for this report were not considered in calculating the averages for *Spinacea oleracea*.

^*e*^ Young *et al.* (2015) consists of only two measurement temperatures (0 and 20 ºC); $Q_{10}^{{}^{35-25}}$ and $Q_{10}^{{}^{45-35}}$ were not calculated.

^*f*^ Low adjustment of the Excel Solver; *c* and ∆*H*_a_ of this report were not considered for group averages.

^*g*^ *r* is not provided given the large scattering between measured *vs*. predicted values.

Analogous simulations were conducted to compare the average C_3_ *in vitro* temperature response functions developed here and three different temperature functions widely used in the literature in simulating photosynthesis, i.e. *in vivo* Rubisco temperature responses for *Nicotiana tabacum* (Bernacchi *et al.*, 2001; Walker *et al.*, 2013) and *in vitro* Rubisco temperature responses for *Spinacia oleracea* (Jordan and Ogren, 1984). To quantitatively compare different simulated temperature response curves, warm *vs*. cool C_3_ plants and temperature response curves currently in use in the modeling community, mean absolute (σ_A_) and root mean squared (σ_S_) differences between different model estimates (Willmott and Matsuura, 2005; Niinemets *et al.*, 2013) were calculated through the modeled temperature range of 5–50 ºC. The mean absolute difference was calculated as:

$$\sigma_{\text{A}}=\frac{1}{n}\sum_{\text{i}=1}^{n}\left|A_{\text{Rubisco,f1}}\left(T_{\text{i}}\right)-A_{\text{Rubisco,f2}}\left(T_{\text{i}}\right)\right|,$$ (15)

where *A*_Rubisco,f1_(*T*_i_) is the estimated *A*_Rubisco_ for the first function at temperature *T*_i_ and *A*_Rubisco,f2_(*T*_i_) is the corresponding *A*_Rubisco_ value for the second function. The root mean squared differences was further calculated as:

$$\sigma_{\text{S}}=\sqrt{\frac{1}{n}\sum_{\text{i}=1}^{n}{\left[A_{\text{Rubisco,f1}}\left(T_{\text{i}}\right)-A_{\text{Rubisco,f2}}\left(T_{\text{i}}\right)\right]}^{2}}.$$ (16)

### Statistical analysis and tests for phylogenetic signals and trait correlations

Conventional statistical analyses consisted of one-way ANOVA and correlation and linear regression analyses. For all the parameters studied, a univariate model of fixed effects was assumed. The univariate general linear model for unbalanced data (Proc. GLM) was applied and significant differences among groups of species were revealed by Duncan’s test. To avoid type II errors due to limited data, only groups with at least five species were statistically compared. In particular, the limited data available on the *in vitro* temperature dependence of *K*_o_ impeded the comparative analysis among groups of species for temperature responses of this characteristic. Modeled temperature responses of *A*_Rubisco_ were compared by pairwise *t*-test over temperature ranges of 5–20 and 30–50 ºC. In addition, paired *t*-tests were used to compare the mean absolute and root mean squared differences in model estimates among different groups of model datasets (*in vitro* warm *vs*. cool C_3_ dataset developed in this study *vs*. three currently widely used model datasets, comparisons conducted for four different simulations with chloroplastic CO_2_ concentrations of 120, 150, 200 and 400 μmol mol^−1^). These analyses were conducted with the IBM SPSS Statistics 20 software package (IBM, Armonk, NY, USA).

In order to test phylogenetic signal strength on trait correlations (the theoretical background of these analyses followed the framework set in Galmés *et al.* (2015)), complete phylogeny was assembled for all the species in this study. For this, we used *RbcL* and 16S ribosomal RNA (for species with no available *RbcL*) sequences from GenBank (http://www.ncbi.nlm.nih.gov). Where genetic data were not available for the given species, we obtained data from GenBank for functionally similar species from the same genus that had overlapping distribution. Phylogeny was constructed in MEGA6 (Tamura *et al.*, 2013), using standardized methods of aligning multiple sequences: Muscle (Edgar, 2004) and constructing maximum likelihood phylogenetic tree (Chor and Tuller, 2005). Phylogenetic independent contrasts, indicative of the strength of phylogenetic signal, were calculated in R using packages ‘ape’ (Paradis *et al.*, 2004), ‘nlme’ (Pinheiro *et al.*, 2014) ‘geiger’ (Harmon *et al.*, 2008) and ‘phytools’ (Revell, 2012). The effects of phylogenetic signal on trait correlations were assessed by analysis of covariance (ANCOVA) and by calculating Pagel’s lambda (λ) based on phylogenetic independent contrasts values (Pagel, 1999). All statistical differences were considered significant at *P*<0.05.

## Results

### Standardization of *in vitro* data

The default value of acidity constant of dissolved CO_2_, $\text{p}K_{{\text{a,CO}}_{2}}$, for pure water used in early studies was commonly taken as 6.35–6.37 at 25 °C (with reference to e.g. Harned and Bonner, 1945; Umbreit *et al.*, 1972). Across the studies, the average ionic strength of the assay medium (*I*_s_) at 25 ºC was 0.117±0.006M (range 0.066–0.165M), and the predicted true value of $\text{p}K_{{\text{a,CO}}_{2}}$ (Eq. 2) corresponding to this average estimate of *I*_s_ is 6.112, while the equation of Yokota and Kitaoka (1985) suggests a value of 6.118. Given further that the average pH used in *K*_c_ assays at 25 ºC was 8.17±0.04 (range 8.0–8.5), using the $\text{p}K_{{\text{a,CO}}_{2}}$ estimates of pure water would overestimate *K*_c_ by 1.75-fold (and 1.015-fold for the equation of Yokota and Kitaoka (1985)). At 35 °C, the $\text{p}K_{{\text{a,CO}}_{2}}$ for pure water is 6.32 (Harned and Bonner, 1945), while the predicted true value for the average *I*_s_ is 6.067 (predicted overestimation by 1.78-fold), and the value predicted according to Yokota and Kitaoka (1985) is 6.076 (predicted overestimation 1.021-fold). In our analysis, across all data (different species and temperatures pooled) the average error (±SE) in *K*_c_ estimates (*K*_c,c_ for standardized and *K*_c,m_ for measured *K*_c_ values), 100(*K*_c,c_–*K*_c,m_)/*K*_c,m_ was −19.8±1.6% (range −50 to 10%). For comparison, the overall variation in *K*_c,c_ values across species and temperatures was 206-fold (average±SE=31±5 μM, coefficient of variation of 179%).

In the case of *K*_o_, differences among the estimates can result both from the effects of solutes on *H*_pc_ for oxygen and from differences in *H*_pc,0_ values used among the studies to estimate O_2_ concentration in solutions (Eqs 4–6), while differences in *S*_c/o_ can include both differences in $\text{p}K_{{\text{a,CO}}_{2}}$ and oxygen solubility calculations (Eq. 7). Equations 4 and 5 predict an $H_{{\text{pc,s,O}}_{2}}$ value of 83950 Pa m^3^ mol^−1^ at 25 ºC for the average ion concentration observed in our study, while the typical value of *H*_pc,0,O2_ used in original studies was 80040 Pa m^3^ mol^−1^. As Eq. 6 indicates, the use of the default value of Henry’s law constant would lead to 4.9% underestimation in *K*_o_ at 25 ºC. In our database, across all species and temperature combinations, the average error (±SE) in *K*_o_ estimates was −4.6±0.3% (range −2.4 to −6.6%), whereas the variation in standardized *K*_o_ values across species and temperatures was 28-fold (average±SE=560±100mM, coefficient of variation of 119%). In the case of *S*_c/o_ where both bicarbonate equilibrium and O_2_ solubility play a role, average estimate deviation for all species and temperature combination was −3.5±0.7% (range −10.2 to 66.3%), whereas the variation in standardized *S*_c/o_ values across species and temperatures was 32-fold (average±SE=92.3±2.6mol mol^−1^, coefficient of variation of 39%).

### Comparison of different functions in capturing the Rubisco temperature responses

Both the exponential and the polynomial functions used to fit the temperature responses of Rubisco characteristics (Eqs 12–14) provided a good fit to the data, with most *r* values (for predicted *vs*. measured trait values) for individual relationships greater than 0.950 (Tables 2 and 4, and data not shown). With some exceptions indicated in Table 2, differences between *r* values from second- and third-order polynomial equations (Eqs 13–14) and the Arrhenius-type function (Eq. 12) were minor (for instance, average *r* values for *in vitro K*_o_ were 0.951, 0.974 and 0.913, respectively). For all kinetic parameters, significant correlation was found between polynomial- and Arrhenius-derived values of $Q_{10}^{{}^{25–15}}$ and $Q_{10}^{{}^{35–25}}$ (data not shown). To the contrary, the relationship between polynomial- and Arrhenius-derived values of $Q_{10}^{{}^{15–5}}$ and $Q_{10}^{{}^{45–35}}$ was non-significant for some kinetic parameters. This fact indicates that second- and especially third-order polynomial equations are problematic in predicting kinetics values out of the range of assayed temperatures. Therefore, we suggest using the Arrhenius-type function in capturing the Rubisco temperature responses.

### Overall variability of the Rubisco *in vitro* temperature response parameters for *S*_c/o_, *K*_c_ and *K*_o_

Among all 38 species, the energy of activation (∆*H*_a_) for *S*_c/o_ ranged between −43.0 kJ mol^−1^ for the *Thermosynechococcus elongatus* and −11.7 kJ mol^−1^ for *Synechococcus lividus* (Table 2). With regard to *K*_c_, *Zea mays* had the lowest (∆*H*_a_=22.9 kJ mol^−1^) and *Oryza sativa* the highest (∆*H*_a_=58.4 kJ mol^−1^) temperature-dependent increases in *K*_c_ among the 20 species (Table 2). For both *S*_c/o_ and *K*_c_, a high correlation was observed between ∆*H*_a_ and the *Q*_10_ coefficients calculated at specific ranges of measurement temperatures (*r*>0.995), so that the extremes of the range for *Q*_10_ were generally represented by the same species as for ∆*H*_a_ (Table 2).

The *in vitro* temperature response of *K*_o_ is the least documented trait of Rubisco with data only available for five land plant species (Table 2). Moreover, the values of the temperature dependence parameters for these five species are contradictory. Hence, Badger and Collatz (1977) for *Atriplex glabriuscula*, and Lehnherr *et al.* (1985) for *Trifolium repens* reported positive values of ∆*H*_a_ for *K*_o_ (i.e. *K*_o_ increasing with temperature of measurement); Laing *et al.* (1974) for *Glycine max*, and Boyd *et al.* (2015) for *Setaria viridis* reported negative values of ∆*H*_a_ for *K*_o_ (i.e. *K*_o_ decreasing with increases of the temperature of measurement); and Jordan and Ogren (1984) reported that *Spinacia oleracea K*_o_ was insensitive to temperature of measurement (∆*H*_a_=0 kJ mol^−1^). Data standardization did not change the direction of *K*_o_ temperature responses in any of the cases.

### Comparison of the *in vitro* temperature response parameters for *S*_c/o_, *K*_c_ and $k_{cat}^{c}$ among groups of species

**Fig. 1.**
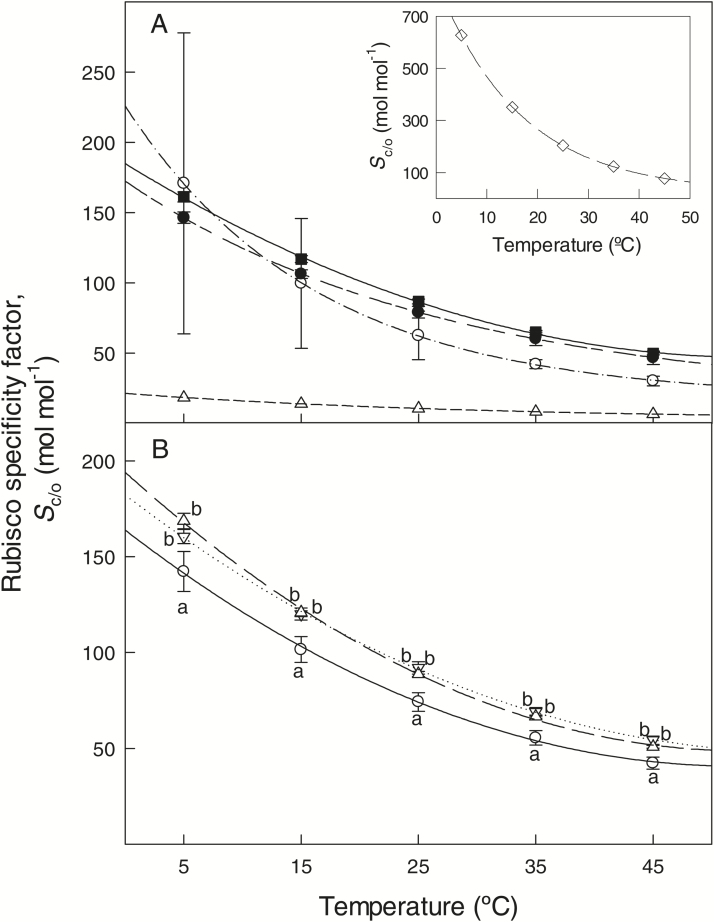
Values of the Rubisco specificity factor for CO_2_/O_2_ (*S*_c/o_) in liquid phase at a range of temperatures in different phylogenetic groups of photosynthetic organisms (A) and in land plants only (B). (A) Open upward triangles and short-dashed line, Proteobacteria; open circles and dash-dotted line, Cyanobacteria; filled circles and long-short-dashed line, Bacillariophyta (diatoms); empty diamond and long-dashed line, Rhodophyta (red algae); filled squares and solid line, Spermatophyta (plants). Sample number *n*=4 for Bacillariophyta and *n*=30 for Spermatophyta; no replication was available for Proteobacteria, Cyanobacteria and Rhodophyta. The inset in shows the values of *S*_c/o_ for Rhodophyta. (B) Open downward triangles and dotted line, C_3_ plants from cool habitats (*n*=8); open upward triangles and long-dashed line, C_3_ plants from warm habitats (*n*=16); open circles and solid line, C_4_ plants (*n*=6). Different letters denote statistically significant differences by Duncan’s analysis (*P*<0.05) among plant functional and photosynthetic groups. All data for *S*_c/o_ correspond to *in vitro* measurements at discrete temperatures from data shown in Table 3 after applying Eq. 12, and were standardized to a common set of liquid-phase CO_2_ and O_2_ physico-chemical characteristics by Eqs 1–7. For CO_2_, these equations correct for study-to-study differences in assumed bicarbonate equilibrium as dependent on solution pH, temperature and ionic strength and when pertinent study-to-study differences in the value of Henry’s law constant used. For O_2_, these equations standardize for differences in the value of Henry’s law constant used. Means and standard errors are provided when *n*≥2. Table 1 provides Henry’s law constants that can be used to convert the Rubisco kinetic characteristics to gas-phase equivalent values.

Across all the measurement temperatures analysed, average *S*_c/o_ values for Spermatophyta were lower than those for Rhodophyta (*Galdieria partita*), higher than those for Proteobacteria (*Rhodospirillum rubrum*), and similar to Bacillariophyta and Cyanobacteria (Fig. 1A). The divergence between the two Cyanobacteria species, *Synechococcus lividus* and *Thermosynechococcus elongates*, resulted in large standard errors at ≤25 ºC for this group. In all phylogenetic groups, Rubisco *S*_c/o_ decreased with increasing the assay temperature, but the extent of such decrease differed among groups. Hence, average ∆*H*_a_ for *S*_c/o_ of Spermatophyta (−21.5 kJ mol^−1^) was similar to the values reported for Proteobacteria and Bacillariophyta, and 44% lower compared with Rhodophyta (in absolute values, Table 3). The comparison of average ∆*H*_a_ for *S*_c/o_ of Spermatophyta with that of Cyanobacteria depended on the species (1.8-fold higher compared with *Synechococcus lividus* and 50% lower compared with *Thermosynechococcus elongatus*).

**Table 3.** Average temperature dependence parameters of the *in vitro*-measured Rubisco specificity factor for CO_2_/O_2_ (*S*_c/o_), the Michaelis–Menten constant for CO_2_ (*K*_c_) and the Rubisco maximum carboxylase turnover rate ($k_{\text{cat}}^{\text{c}}$)

Group	*n*	*c*	∆*H*_a_ (kJ mol^−1^)	$Q_{10}^{{}^{15–5}}$	$Q_{10}^{{}^{25–15}}$	$Q_{10}^{{}^{35–25}}$	$Q_{10}^{{}^{45–35}}$	*T*_growth_ (ºC)
*S*_c/o_								
Proteobacteria	1	−5.2	−18.8	0.75	0.77			33
Cyanobacteria	2	−6.9±6.0	−27.4±15.6	0.68±0.16	0.70±0.15	0.71±0.14	0.73±0.14	50.5±5.5
Rhodophyta	1	−10.3	−38.7	0.56	0.58	0.60	0.62	45
Bacillariophyta	4	−4.2±1.0	−21.3±2.3	0.73±0.02	0.74±0.02	0.76±0.02	0.77±0.02	7.8±4.6
Spermatophyta	30	−4.2±0.2	−21.5±0.4	0.73±0.01	0.74±0.01	0.75±0.01	0.77±0.01	25.0±0.7
Spermatophyta (C_3_ plants)	24	−4.1±0.2	−21.4±0.4	0.73±0.01	0.75±0.01	0.76±0.01	0.77±0.01	24.0±0.8
Spermatophyta (C_3_ plants from cool habitats)	8	−3.5±0.3^b^	−19.9±0.6^b^	0.74±0.01^b^	0.77±0.01^b^	0.78±0.01^b^	0.79±0.01^b^	19.5±0.5^a^
Spermatophyta (C_3_ plants from warm habitats)	16	−4.4±0.2^a^	−22.0±0.5^ab^	0.72±0.01^a^	0.74±0.01^a^	0.75±0.01^a^	0.76±0.01^a^	26.3±0.6^b^
Spermatophyta (C_4_ plants)	6	−4.8±0.5^a^	−22.3±1.2^a^	0.72±0.01^a^	0.73±0.01^a^	0.75±0.01^a^	0.76±0.01^a^	29.2±0.8^c^
*K*_c_								
Cyanobacteria	1	20.8	38.8	1.79	1.72	1.66	1.61	35
Bacillariophyta	2	19.4±1.7	39.0±4.1	1.80±0.11	1.73±0.10			13.5±8.5
Spermatophyta	18	19.2±0.9	41.1±2.3	1.85±0.06	1.77±0.06	1.70±0.05	1.64±0.05	25.3±1.1
Spermatophyta (C_3_ plants)	12	20.0±0.8	43.8±2.2	1.91±0.07	1.82±0.06	1.75±0.06	1.68±0.06	23.9±1.4
Spermatophyta (C_3_ plants from cool habitats)	6	18.8±1.2^a^	40.5±3.2^ab^	1.79±0.09^a^	1.71±0.09^a^	1.64±0.09^a^	1.58±0.08^a^	19.3±0.7^a^
Spermatophyta (C_3_ plants from warm habitats)	7	20.9±1.1^a^	46.1±2.8^b^	2.01±0.09^a^	1.92±0.08^a^	1.84±0.07^a^	1.77±0.06^a^	27.9±1.0^b^
Spermatophyta (C_4_ plants)	5	17.2±2.0^a^	34.7±4.7^a^	1.70±0.13^a^	1.64±0.11^a^	1.59±0.10^a^	1.54±0.09^a^	29.0±1.0^b^
$k_{\text{cat}}^{\text{c}}$								
Archaea	1	15.2	37.2	1.75	1.68	1.63	1.58	85.0
Proteobacteria	4	18.5±1.5	45.9±4.1	2.00±0.13	1.91±0.11	1.83±0.10	1.76±0.09	33.8±5.5
Cyanobacteria	3	16.3±3.5	40.1±8.9	1.86±0.26	1.78±0.23	1.71±0.21	1.66±0.19	46.7±7.3
Rhodophyta	1	30.8	76.3	3.14	2.91	2.71	2.55	57.0
Chlorophyta	4	10.8±0.4	26.7±0.9	1.49±0.02	1.45±0.02	1.42±0.02	1.39±0.02	15.5±5.5
Spermatophyta	36	23.5±0.7	58.1±1.7	2.43±0.07	2.28±0.06	2.16±0.05	2.06±0.05	24.9±0.9
Spermatophyta (C_3_ plants)	26	24.3±0.9	60.2±2.3	2.51±0.09	2.35±0.08	2.23±0.07	2.11±0.06	23.1±1.0
Spermatophyta (C_3_ plants from cool habitats)	12	22.3±0.8^a^	55.3±2.0^a^	2.30±0.07^a^	2.18±0.06^a^	2.07±0.05^a^	1.98±0.05^a^	18.3±0.8^a^
Spermatophyta (C_3_ plants from warm habitats)	14	26.0±1.4^b^	64.5±3.5^b^	2.68±0.15^b^	2.51±0.13^b^	2.36±0.11^b^	2.23±0.10^b^	27.2±0.7^b^
Spermatophyta (C_4_ plants)	10	21.3±0.5^a^	52.8±1.3^a^	2.22±0.05^a^	2.10±0.04^a^	2.00±0.03^a^	1.91±0.03^a^	29.6±0.4^b^

The original data for *S*_c/o_ and *K*_c_ were taken from Table 2 and those for $k_{cat}^{c}$ from Galmés *et al.* (2015). For *Spinacea oleracea*, *Triticum aestivum* and *Flaveria pringlei*, average values from Table 2 were used. The values are means±SE, except when *n*=1. Within Spermatophyta, significant differences among C_3_-cool, C_3_-warm and C_4_ species (*P*<0.05 according to one-way ANOVA followed by Duncan’s test) are denoted by different letters. The optimum growth temperature (*T*_growth_) for each species is shown in Table 2. *c*, scaling constant; ∆*H*_a_, activation energy; *Q*_10_ coefficient over the temperature intervals of 5–15 ºC ($Q_{10}^{{}^{15-5}}$), 15–25 ºC ($Q_{10}^{{}^{25-15}}$), 25–35 ºC ($Q_{10}^{{}^{35-25}}$) and 35–45 ºC ($Q_{10}^{{}^{35-25}}$).

Within Spermatophyta, C_4_ plants had lower *S*_c/o_ values than C_3_ plants from cool and warm habitats at all assay temperatures (between 5 and 45 ºC), while non-significant differences were found between C_3_ plants from cool and warm habitats (Fig. 1B). The temperature response of *S*_c/o_ also differed within Spermatophyta, with higher thermal sensitivity of *S*_c/o_ (i.e. more negative ∆*H*_a_ and lower *Q*_10_) in C_4_ plants compared with C_3_ plants from cool habitats (Table 3). C_3_ plants from warm habitats presented intermediate values of ∆*H*_a_ for *S*_c/o_, and similar *Q*_10_ values to C_4_ plants (Table 3).

**Fig. 2.**
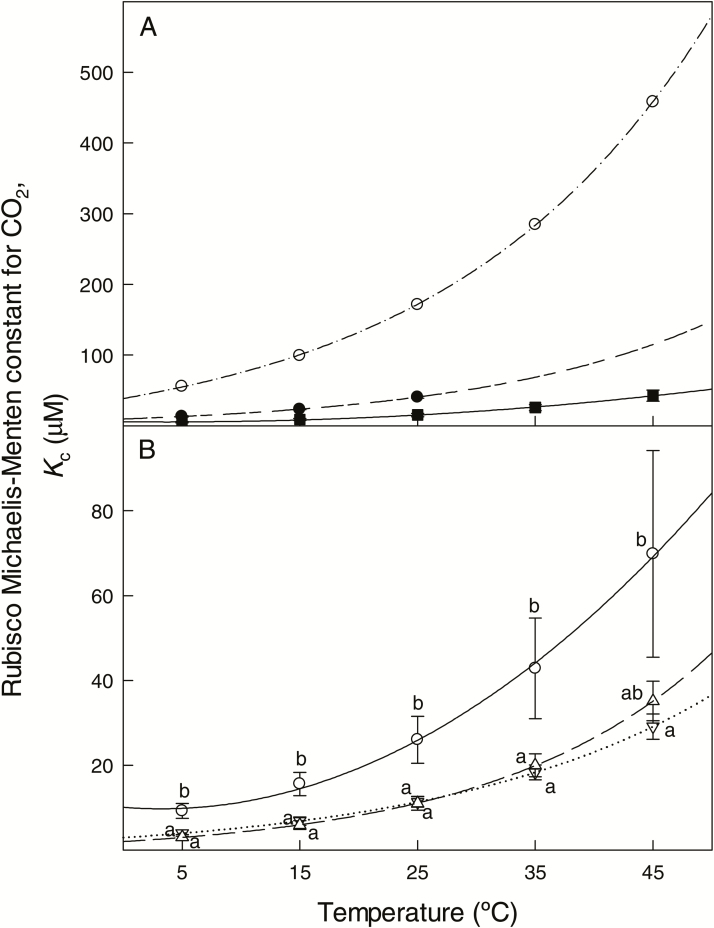
Values of the Rubisco Michaelis–Menten constant for CO_2_ (*K*_c_) in liquid phase at a range of temperatures in different phylogenetic groups (A) and in land plants only (B). (A) Open circles and dash-dotted line, Cyanobacteria; filled circles and dashed line, Bacillariophyta (diatoms); filled squares and solid line, Spermatophyta (plants). Sample number *n*=2 for Bacillariophyta and *n*=17 for Spermatophyta; no replication was available for Cyanobacteria. (B) Open downward triangles and dotted line, C_3_ plants from cool habitats (*n*=5); open upward triangles and dashed line, C_3_ plants from warm habitats (*n*=7); open circles and dotted line, C_4_ plants (*n*=5). Values for *K*_c_ correspond to *in vitro* measurements at discrete temperatures from data shown in Table 3 after applying Eq. 12, and were standardized to a common set of CO_2_ liquid-phase physico-chemical characteristics as explained in Fig. 1 (Table 1 for Henry’s law constants for CO_2_ and O_2_ that can be used to convert the values reported to gas-phase equivalents). Data presentation as in Fig. 1.

Rubisco from Spermatophyta had a higher affinity for CO_2_ (i.e. lower *K*_c_) than Rubiscos from Bacillariophyta and Cyanobacteria throughout the range of temperatures of measurement (Fig. 2A). For the three phylogenetic groups with available data, *K*_c_ values from *in vitro* measurements increased with the temperature of measurement, and the values of ∆*H*_a_ and *Q*_10_ for *K*_c_ were similar among the groups (Table 3). Within land plants, Rubisco from C_4_ plants displayed higher *K*_c_ values compared with Rubiscos from C_3_ plants, at all temperatures of measurement except 45 ºC (Fig. 2B). Differences in *K*_c_ between C_3_ plants from cool and warm habitats were non-significant across the range of temperatures of measurement. The temperature dependence of *K*_c_ varied within Spermatophyta, with Rubisco from C_4_ plants presenting lower values of ∆*H*_a_ compared with C_3_ plants from warm habitats, while differences between C_3_ plants from cool and warm habitats were non-significant (Table 3). Differences in *Q*_10_ for *K*_c_ among higher plants groups were non-significant.

Reanalysing the $k_{\text{cat}}^{\text{c}}$ data compilation of Galmés *et al.* (2015) in terms of *Q*_10_, we note that in the comparisons of the temperature response of $k_{\text{cat}}^{\text{c}}$ among Spermatophyta, Rubisco from C_4_ plants presented lower values of ∆*H*_a_ and *Q*_10_ than C_3_ plants from warm habitats (Table 3). Similarly to the *S*_c/o_ temperature dependence, $k_{\text{cat}}^{\text{c}}$ of C_3_ plants from warm environments was more sensitive to increases in temperature (i.e. higher ∆*H*_a_ or *Q*_10_) than those of C_3_ plants from cool environments (Table 3).

The calculated values for ∆*H*_a_ integrate all data of the temperature response curve, while *Q*_10_ values refer to specific thermal ranges of the curve. With the exception of ∆*H*_a_ *vs*. *Q*_10_ for *K*_c_ in the comparison among higher plant groups, the trends observed for *Q*_10_, in terms of species or groups comparison, are identical to those described for ∆*H*_a_ (Table 3) in all kinetic parameters. Due to the changes in scaling exponent of the temperature response of Rubisco kinetics, values of *Q*_10_ for *S*_c/o_ increase from $Q_{10}^{{}^{15–5}}$ to $Q_{10}^{{}^{45–35}}$, while they decrease for *K*_c_ and $k_{\text{cat}}^{\text{c}}$, in all groups of species.

Phylogenetic signals were not significant in ANCOVA models (*P*-values for *S*_c/o_, *K*_c_ and $k_{\text{cat}}^{\text{c}}$ within Spermatophyta were 0.519, 0.114 and 0.742, respectively). This fact indicates that when corrected for the phylogenetic signal, the comparison of *S*_c/o_ and *K*_c_ values at given temperatures (Figs 1B and 2B), as well as the differences in temperature dependence parameters for *S*_c/o_, *K*_c_ and $k_{\text{cat}}^{\text{c}}$ among Spermatophyta groups (Table 3), were qualitatively identical to the conventional statistics (ANOVA).

### Relationship between the energies of activation of Rubisco catalytic traits and the species optimum growth temperature

**Fig. 3.**
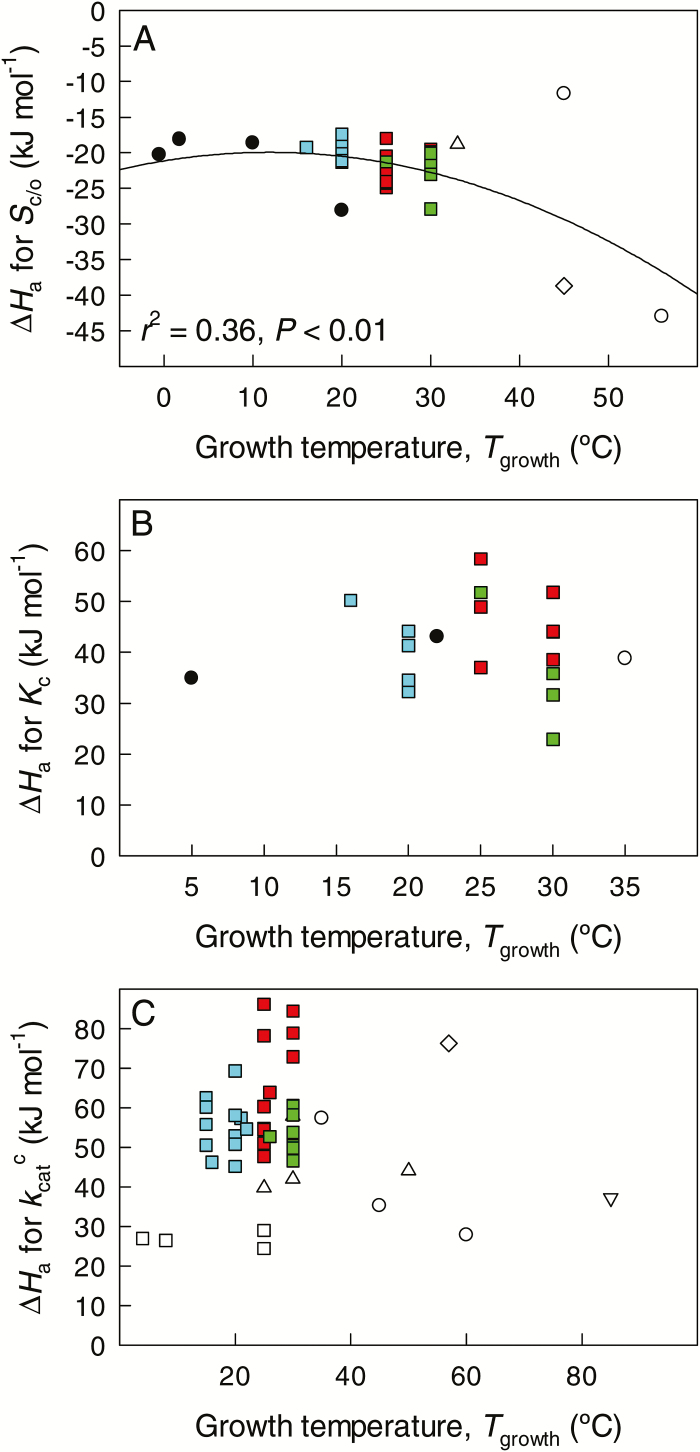
The relationship between the growth temperature (*T*_growth_) and the energy of activation (∆*H*_a_) for (A) the Rubisco specificity factor for CO_2_/O_2_ (*S*_c/o_) in liquid phase, (B) the Rubisco Michaelis–Menten constant for CO_2_ (*K*_c_) in liquid phase, and (C) the Rubisco maximum carboxylase turnover rate ($k_{cat}^{c}$). Each symbol corresponds to individual species (Table 2 for data sources). Open upward triangles, Proteobacteria; open circles, Cyanobacteria; black circles, Bacillariophyta (diatoms); open squares, Chlorophyta (green algae); open diamond, Rhodophyta (red algae); blue squares, C_3_ plants from cool habitats; red squares, C_3_ plants from warm habitats; green squares C_4_ plants. *In vitro* estimates at discrete temperatures were standardized for study-to-study differences in physico-chemical characteristics for CO_2_ and O_2_ used as in Figs 1 and 2 and the temperature responses were fitted by Eq. 12. In (A), the data were fitted by a non-linear equation in the form *y*=−20.911+0.207*x*–0.009*x*^2^. In (B) and (C), data fits by linear and different monotonic non-linear equations were statistically not significant (best *r*^2^ values were 0.090 for (B) and 0.115 for (C), *P*>0.1 for both).

An inverse relationship was found between ∆*H*_a_ for *S*_c/o_ and the species average optimum growth temperature (*T*_growth_), suggesting that *S*_c/o_ values of Rubisco from species inhabiting hot environments present a higher sensitivity to changes in temperature (Fig. 3A). Although this relationship was significant and not affected by the species’ phylogeny (Pagel’s λ=0.575), it was substantially influenced by the values of the thermophiles *Thermosynechococcus elongatus* (Cyanobacteria) and *Galdieria partita* (Rhodophyta), which presented the highest *T*_growth_ and the lowest ∆*H*_a_ for *S*_c/o_. Furthermore, *Synechococcus lividus*, with the same *T*_growth_ as *Galdieria partita* (45 ºC), had the highest ∆*H*_a_ for *S*_c/o_ among all the species studied. Nevertheless, when the data were reanalysed without these three species with *T*_growth_>40 ºC, there was still a significant correlation between ∆*H*_a_ for *S*_c/o_ and *T*_growth_ (*r*^2^=0.175, *P*<0.02 for a second-order polynomial regression). Clearly, there is evidence of common trends in temperature scaling of *S*_c/o_ across disparate phylogenetic groups, indicating that convergent evolution has led to similar functional responses. In contrast, the relationships between *T*_growth_ and ∆*H*_a_ for *K*_c_ and $k_{\text{cat}}^{\text{c}}$ were non-significant (Figs 3B, C). The relationships were analogous with *Q*_10_ values (data not shown).

**Fig. 4.**
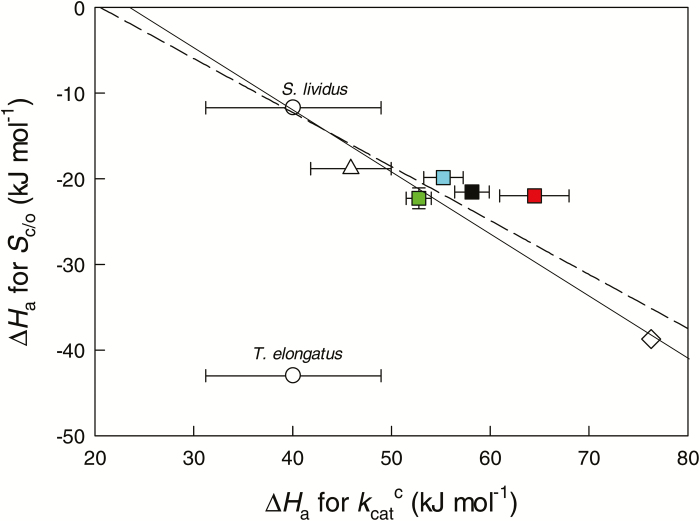
The relationship between the energies of activation (∆*H*_a_) for the Rubisco maximum carboxylase turnover rate ($k_{cat}^{c}$) and the Rubisco specificity factor for CO_2_/O_2_ (*S*_c/o_) in liquid phase across domains of life and photosynthetic and ecological groupings of plants (symbols as in Fig. 3). Data were separately fitted by linear regressions across domains of life (all plants averaged; solid line, *r*^2^=0.952, *P*<0.01) and across all groupings (plant functional and photosynthetic groupings, C_3_ cool and warm and C_4_, separately considered; dashed line, *r*^2^=0.846, *P*<0.01). In Rhodophyta, the value of ∆*H*_a_ for *S*_c/o_ is from *Galdieria partita*, while that of ∆*H*_a_ for $k_{cat}^{c}$ is from *Cyanidium caldarium*. For the other phylogenetic groups, data correspond to averages±SE from different numbers of species (Table 3 for data sources). Data for *Thermosynechococcus elongatus* (Cyanobacteria) with vastly different Rubisco kinetics (Figs 1 and 2) were not considered in the regression analysis.

When considering group averages, a negative relationship was found between ∆*H*_a_ for *S*_c/o_ and ∆*H*_a_ for $k_{\text{cat}}^{\text{c}}$ in all groups except the Cyanobacterium *T. elongatus* (Fig. 4). This relationship was significant both when all plants were averaged and when plant functional and photosynthetic groupings were separately considered, and was not affected by the phylogenetic signal (Pagel’s λ=−0.936, and ANCOVA *P*=0.21, respectively).

### Temperature dependence of *in vivo*-estimated Rubisco kinetics and the relationship with the temperature parameters derived from *in vitro* measurements

**Fig. 5.**
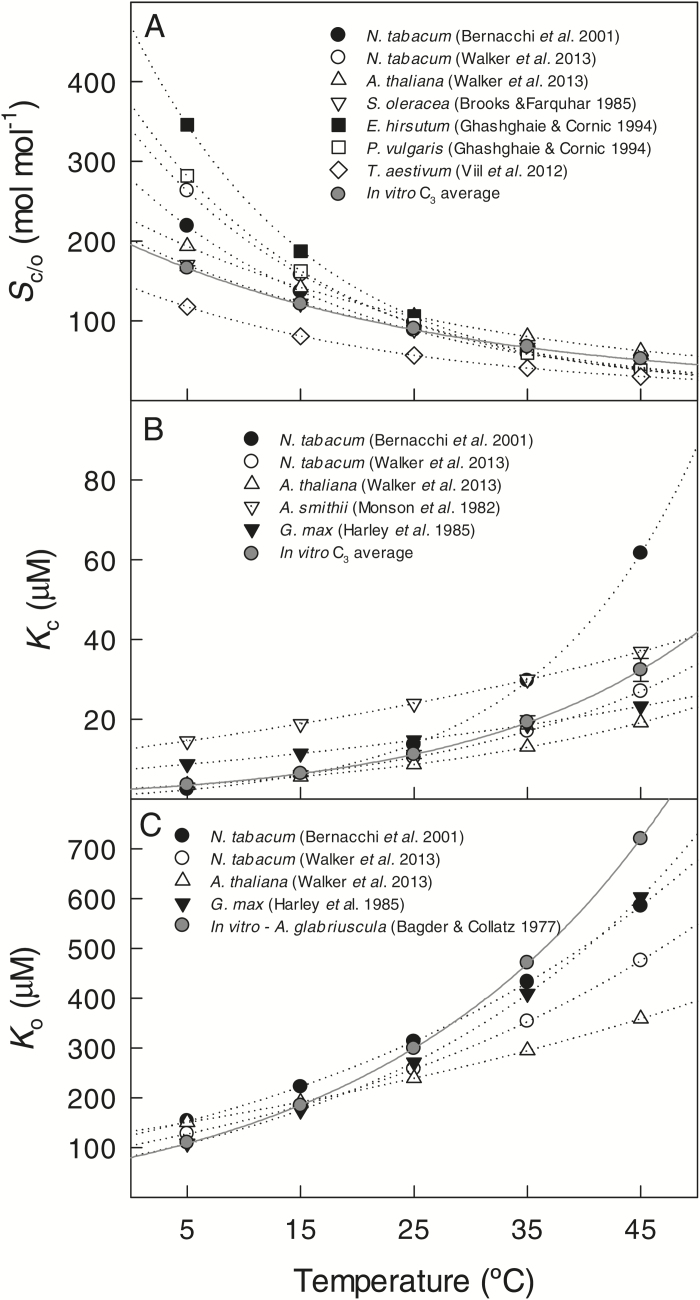
Values of the Rubisco specificity factor for CO_2_/O_2_ (*S*_c/o_) (A) and the Michaelis–Menten constants for CO_2_ (*K*_c_) (B) and O_2_ (*K*_o_) (C) at a range of temperatures. Values for these parameters were obtained at discrete temperatures from *in vivo* gas-phase data (shown in Table 4) after applying Eq. 12 and converted to the liquid phase by Eqs 8–10 (Table 1 for corresponding Henry’s law constants to convert between liquid-phase and gas-phase equivalents). For comparative purposes, *in vitro* C_3_ average values for *S*_c/o_ and *K*_c_ have been also included (using data shown in Table 3). In (C), *in vitro K*_o_ data for *Atriplex glabriuscula* (Badger and Collatz 1977, shown in Table 2) that have been widely used to model leaf photosynthesis (see Introduction) have been also included.

In the six species with available data, *in vivo*-estimated *S*_c/o_ decreased with increasing temperature, although important differences existed among the species in the rate of decrease (Fig. 5A). As a consequence, ∆*H*_a_ for *S*_c/o_ varied 2-fold between *Arabidopsis thaliana* (−20 kJ mol^−1^) and *Epilobium hirsutum* (−40.8 kJ mol^−1^; Table 4). The average values of *in vitro S*_c/o_ for C_3_ plants fell within the lower range of the *in vivo*-estimated values, and the *in vitro*-based ∆*H*_a_ for *S*_c/o_ (−21.4 kJ mol^−1^, Table 3) was similar to that estimated *in vivo* for *A. thaliana* and *S. oleracea* (Fig. 5A).

**Table 4.** Temperature dependence parameters of *in vivo*-measured Rubisco catalytic constants for Spermatophyta

Group	Species	Reference	*T*_meas_ (ºC)	*c*	∆*H*_a_ (kJ mol^−1^)	*r*	$Q_{10}^{{}^{15–5}}$	$Q_{10}^{{}^{25–15}}$	$Q_{10}^{{}^{35–25}}$	$Q_{10}^{{}^{45–35}}$	*T*_growth_ (ºC)
*S*_c/o_											
C_3_ plants from cool habitats	*Epilobium hirsutumSpinacia oleracea* hybrid 102	Ghashghaie and Cornic (1994)Brooks and Farquhar (1985)	16–2815–30	−11.8−4.5	−40.8−22.3	0.9780.993	0.540.72	0.570.73	0.590.75	0.610.76	18.016.0
	*Triticum aestivum* cv. Saratovskaya 29	Viil *et al.* (2012)	5–41	−6.1	−25.2	0.997	0.69	0.70	0.72	0.73	20.0
	*Arabidopsis thaliana* cv. Columbia	Walker *et al.* (2013)	15–35	−3.8	−20.9	0.956	0.73	0.75	0.76	0.77	22.0
C_3_ plants from warm habitats	*Nicotiana tabacum* cv. W38	Bernacchi *et al.* (2001)	10–40	−8.2	−31.4	0.997	0.62	0.64	0.66	0.68	25.0
	*Nicotiana tabacum*	Walker *et al.* (2013)	15–35	−9.2	−34.2	0.950	0.60	0.62	0.64	0.66	25.0
	*Phaseolus vulgaris*	Ghashghaie and Cornic (1994)	12–32	−10.3	−36.8	0.973	0.58	0.60	0.62	0.64	25.0
*K*_c_											
C_3_ plants from cool habitats	*Arabidopsis thaliana* cv. Columbia	Walker *et al.* (2013)	15–35	14.8	31.4	0.946	1.60	1.55	1.51	1.47	22
C_3_ plants from warm habitats	*Agropyron smithiiGlycine max* cv. P61–22	Monson *et al.* (1982)	10–40	10.1	17.1	0.933	1.29	1.27	1.25	1.23	25
		Harley *et al.* (1985)	20–40	10.0	18.0	0.993	1.31	1.29	1.27	1.25	25
	*Nicotiana tabacum* cv. W38	Bernacchi *et al.* (2001)	10–40	26.6	59.5	0.995	2.44	2.30	2.18	2.07	25
	*Nicotiana tabacum*	Walker *et al.* (2013)	15–35	17.5	37.6	0.991	1.76	1.69	1.64	1.59	25
*K*_o_											
C_3_ plants from cool habitats	*Arabidopsis thaliana* cv. Columbia	Walker *et al.* (2013)	15–35	11.9	16.0	0.734	1.27	1.25	1.23	1.22	22
C_3_ plants from warm habitats	*Nicotiana tabacum* cv. W38	Bernacchi *et al.* (2001)	10–35	15.7	24.6	0.991	1.45	1.41	1.38	1.35	25
	*Nicotiana tabacum*	Walker *et al.* (2013)	15–35	15.3	24.1	0.935	1.44	1.40	1.37	1.34	25
	*Glycine max* cv. P61–22	Harley *et al.* (1985)	20–40	18.3	31.5	0.988	1.60	1.55	1.51	1.47	25

Species were classified as C_3_ and C_4_ species, and C_3_ species were further divided among warm- and cool-temperature species according to their optimum growth temperature (Table 2). *c*, scaling constant; ∆*H*_a_, activation energy; *K*_c_, Michaelis–Menten constant for CO_2_; *K*_o_, Michaelis–Menten constant for O_2_; *Q*_10_, coefficient over the temperature intervals of 5–15 ºC ($Q_{10}^{{}^{15-5}}$), 15–25 ºC ($Q_{10}^{{}^{25-15}}$), 25–35 ºC ($Q_{10}^{{}^{35-25}}$) and 35–45 ºC ($Q_{10}^{{}^{45-35}}$); *r*, correlation coefficient for linear regressions between measured *vs*. predicted (Microsoft Excel Solver function) values of each kinetic parameter at the assayed temperatures; *S*_c/o_, specificity factor for CO_2_/O_2_; *T*_meas_, range of measurement temperature.

An increase in the *in vivo K*_c_ with increasing temperature of measurement was observed for all the species (Fig. 5B), but the ∆*H*_a_ for *K*_c_ varied three-fold between 17.1 kJ mol^−1^ for *Agropyron smithii* (Monson *et al.*, 1982) and 59.5 kJ mol^−1^ for *Nicotiana tabacum* (Bernacchi *et al.*, 2001) (Table 4). The average (±SE) energy of activation for *in vitro K*_c_ in C_3_ plants was 43.8±2.2 kJ mol^−1^ (Table 3), i.e. higher than all the *in vivo*-based values except that for *N. tabacum* described in Bernacchi *et al.* (2001).

The temperature response of *in vivo*-estimated *K*_o_ has been reported for *N. tabacum*, *A. thaliana* and *Glycine max* (Table 4). In contrast to some *in vitro* data (Table 2), all *in vivo* data exhibited a positive scaling of *K*_o_ with temperature (Fig. 5C). Compared with the *in vivo K*_o_ values, the *in vitro K*_o_ of the C_3_ plant *Atriplex glabriuscula* (Badger and Collatz, 1977) presented lower values at temperatures below 25 ºC and higher values at temperatures above 25 ºC. The higher thermal sensitivity of the *in vitro K*_o_ of *A. glabriuscula* resulted in a higher ∆*H*_a_ for *K*_o_ (34.6 kJ mol^−1^, Table 2) compared with the *in vivo*-based values for ∆*H*_a_ of other plants (Table 4).

In the case of *in vivo* data, only the study of Walker *et al.* (2013) considered the leaf mesophyll conductance, i.e. used the ‘true’ chloroplastic CO_2_ concentration in the derivation of Rubisco characteristics by inverting the FvCB photosynthesis model. Indeed, *S*_c/o_ and *K*_c_ temperature characteristics for *A. thaliana* estimated in this study are closer to C_3_ average *in vitro* data than most of the other estimates (Fig. 5A, B). However, in *N. tabacum*, *S*_c/o_ *in vivo* temperature characteristics estimated without mesophyll conductance in the study of Bernacchi *et al.* (2001) are actually closer to C_3_ average estimates than *N. tabacum in vivo* characteristics estimated by considering mesophyll conductance in the study of Walker *et al.* 2013 (Fig. 5).

**Fig. 6.**
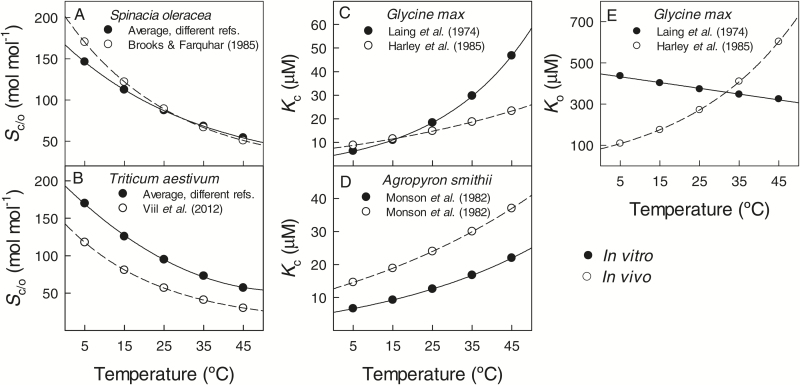
Comparisons between *in vitro* (filled symbols) and *in vivo* (open symbols) values of the Rubisco specificity factor for CO_2_/O_2_ (*S*_c/o_) (A, B) and the Michaelis–Menten constants for CO_2_ (*K*_c_) (C, D) and O_2_ (*K*_o_) (E) at a range of temperatures for species with available data. Equation 12 was used to derive estimates for these parameters at discrete temperatures from data in Tables 2 and 4. The *in vitro* liquid-phase data were standardized for a common set of physico-chemical characteristics of CO_2_ and O_2_ as explained in Figs 1 and 2, while the *in vivo* gas-phase data were converted to the liquid phase as explained in Fig. 5 (Table 1 for pertinent Henry’s law constant to convert between liquid- and gas-phase equivalents). *S*_c/o_ data for *Spinacia oleracea* are averages for the studies Jordan and Ogren (1984), Uemura *et al.* (1997), Zhu *et al.* (1998) and Yamori *et al.* (2006). *S*_c/o_ data for *Triticum aestivum* are averages for studies Haslam *et al.* (2005) and Hermida-Carrera *et al.* (2016).

For several species, the temperature responses of Rubisco characteristics had been reported both *in vitro* and *in vivo*, whereas all these *in vivo* analyses had been conducted without considering mesophyll conductance. As for the temperature response of *S*_c/o_, *in vitro* and *in vivo* data were available for *Spinacia oleracea* and *Triticum aestivum* (Fig. 6A, B). In *S. oleracea*, *in vitro* (average from different references) and *in vivo* (Brooks and Farquhar, 1985) data for *S*_c/o_ were similar over the range of measurement temperatures (Fig. 6A). In *T. aestivum*, *in vitro* values (average from different references) were higher than the *in vivo* ones (Viil *et al.*, 2012) at all temperatures of measurement (Fig. 6B). It should be noted that differently from all other *in vivo* studies, Viil *et al.* (2012) used *in vivo* ^14^CO_2_ leaf uptake for *S*_c/o_ derivation.

*In vitro* and *in vivo* data on the temperature response of *K*_c_ have been published for *Glycine max* and *Agropyron smithii* (Fig. 6C, D). In *G. max*, *in vitro* (Laing *et al.*, 1974) and *in vivo* (Harley *et al.*, 1985) reported data were similar over the temperature range 5–25 ºC, but at higher temperatures of measurement *in vitro* values were higher than *in vivo* values (Fig. 6C). In *A. smithii*, *in vivo* estimations for *K*_c_ were higher than *in vitro* values at all temperatures of measurement (Fig. 6D). *Glycine max* is the unique species for which the temperature response of *K*_o_ has been examined both *in vitro* and *in vivo* (Fig. 6E). Differently from other comparisons between *in vitro* and *in vivo* values for *S*_c/o_ and *K*_c_ that were at least qualitatively similar, the *in vivo* values of *K*_o_ (Harley *et al.*, 1985) increased exponentially with increasing temperature, but the *in vitro* values (Laing *et al.*, 1974) tended to decrease.

### Variation in the temperature responses of Rubisco kinetics: implications for modeling photosynthesis

**Fig. 7.**
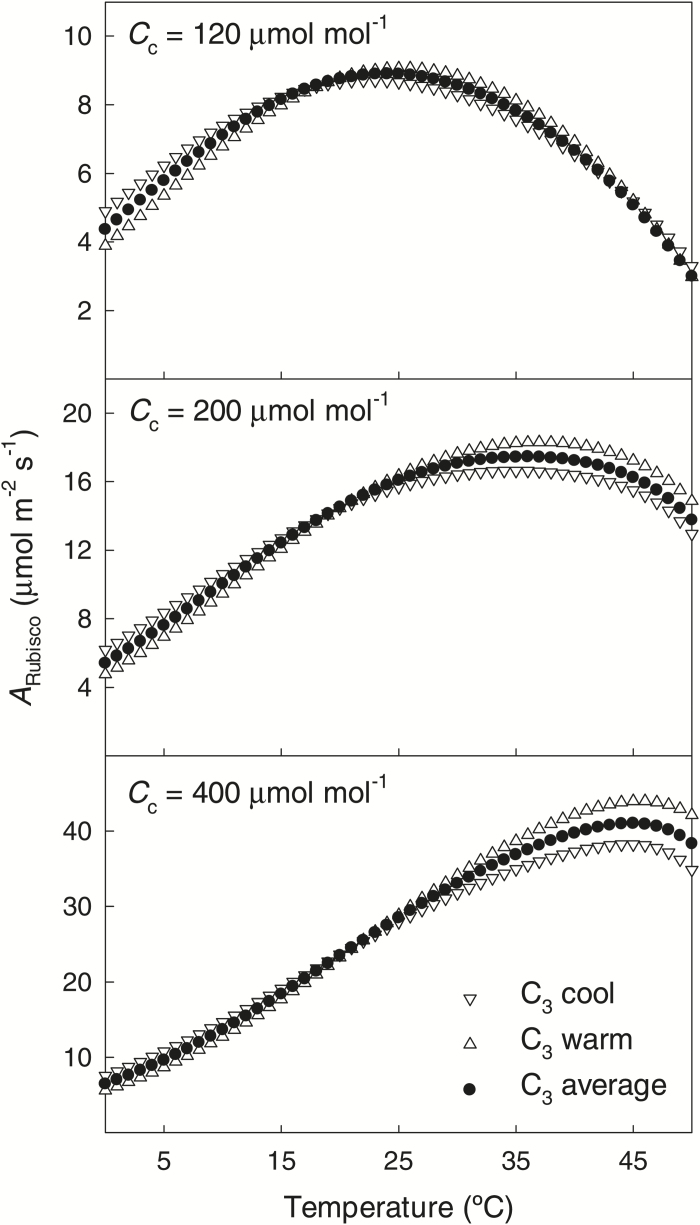
Modeling effect of the different temperature responses of Rubisco kinetic parameters from C_3_ plants from cool habitats (open downward triangles), C_3_ plants from warm habitats (open upward triangles) and C_3_ average (open circles) on the Rubisco-limited gross assimilation rate (*A*_Rubisco_) at chloroplastic CO_2_ concentrations (*C*_c_) of 120, 200 and 400 μmol mol^−1^. To model *A*_Rubisco_ at different temperatures, the values for the temperature dependence parameters of *S*_c/o_, *K*_c_ and $k_{cat}^{c}$ were taken from Table 3 (see Methods for further details). We simulated gross assimilation here to avoid confounding effects of mitochondrial respiration.

The simulation analysis combining all different Rubisco kinetic characteristics indicated that the temperature responses of Rubisco kinetics of C_3_ plants from cool environments resulted in higher simulated Rubisco-limited gross photosynthesis rate (*A*_Rubisco_) at lower temperatures than in C_3_ species from warm environments, while the latter species performed better at higher temperatures (Fig. 7), although the overall differences among the cool and warm C_3_ species were moderate. The mean absolute difference (σ_A_, Eq. 15) between cool and warm C_3_ species was 0.39 μmol m^−2^ s^−1^ at chloroplastic CO_2_ concentration (*C*_c_) of 120 μmol mol^−1^, and 2.9 μmol m^−2^ s^−1^ at *C*_c_=400 μmol mol^−1^, and corresponding root mean squared differences (σ_S_, Eq. 16) were 0.20 μmol m^−2^ s^−1^ at *C*_c_=120 μmol mol^−1^ and 12.5 μmol m^−2^ s^−1^ at *C*_c_=400 μmol mol^−1^.

The predicted quantitative differences in simulated *A*_Rubisco_ between C_3_ plants from cool and warm environments depended on *C*_c_. Hence, at lower temperature, the higher simulated *A*_Rubisco_ for C_3_ plants from cool environments, compared with that for C_3_ plants from warm environments, was more evident at *C*_c_ of 120 μmol mol^−1^ (for the temperature range of 5–20 ºC, average±SE *A*_Rubisco_ was 7.7±0.2 μmol m^−2^ s^−1^ for C_3_ cool *vs*. 7.3±0.3 μmol m^−2^ s^−1^ for C_3_ warm; means are significantly different at *P*<0.001 according to paired samples *t*-test). At higher temperature, the enhancement in simulated *A*_Rubisco_ for C_3_ plants from warm environments was greater at *C*_c_ of 400 μmol mol^−1^ (Fig. 7; for the temperature range of 30–50 ºC, average±SE *A*_Rubisco_ was 36.0±0.4 μmol m^−2^ s^−1^ for C_3_ cool *vs*. 40.8±0.7 μmol m^−2^ s^−1^ for C_3_ warm; means are significantly different at *P*<0.001 according to paired samples *t*-test). Of course, this simulation is only based on two groups of species, and it is further important that there is a significant within group variability in any of the Rubisco temperature traits that is not related to growth temperature (Fig. 3).

**Fig. 8.**
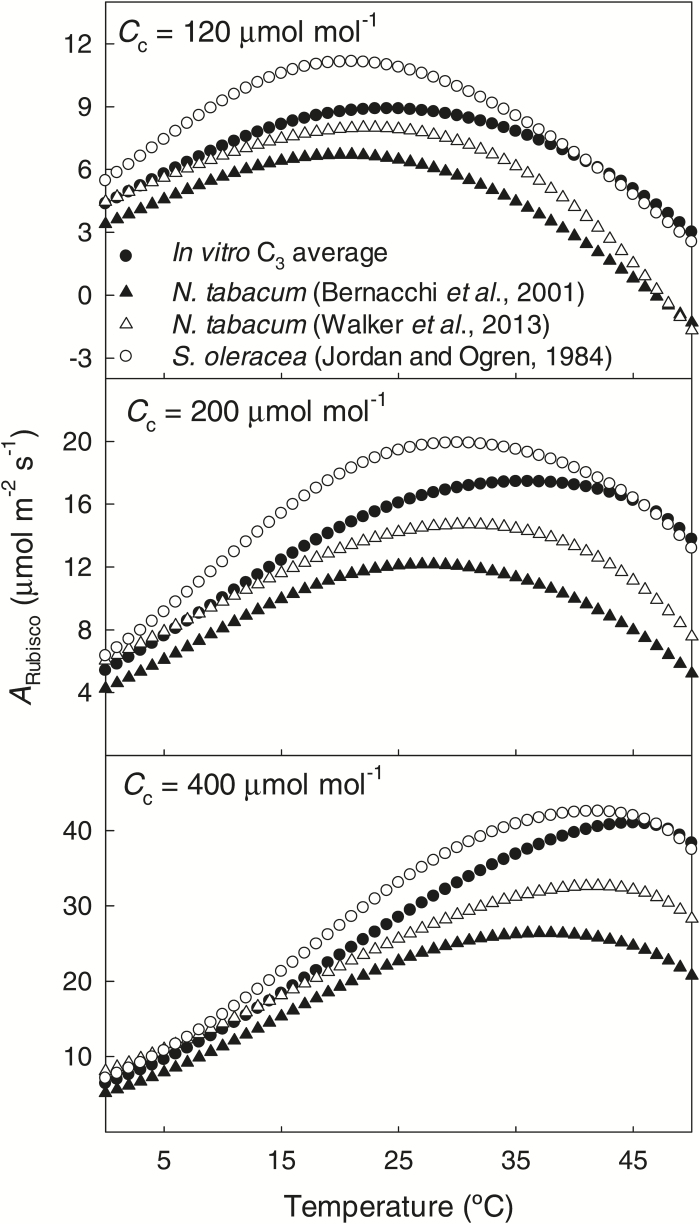
Comparison of the Rubisco-limited gross photosynthesis (*A*_Rubisco_) among average of *in vitro* data reported for C_3_ plants and three widely cited datasets at chloroplastic CO_2_ concentrations (*C*_c_) of 120, 200 and 400 μmol mol^−1^. The temperature dependence parameters of *S*_c/o_, *K*_c_, $k_{cat}^{c}$ and *K*_o_ for *in vitro* average C_3_ plants (shown in Table 3) were the same as in Fig. 7, while those for *in vivo Nicotiana tabacum* (Bernacchi *et al.*, 2001; Walker *et al.*, 2013) and *in vitro Spinacia oleracea* (Jordan and Ogren, 1984) were obtained from the original papers (shown in Tables 2 and 4). Bernacchi *et al.* (2001), Walker *et al.* (2013) and Jordan and Ogren (1984) did not report values of the deactivation energy (∆*H*_d_) and the entropy term (∆*S*) for $k_{cat}^{c}$, and the simulation assumed identical values to those used for the *in vitro* average C_3_ plants (indicated in the Methods). Note that Bernacchi *et al.* (2001) *in vivo* values have been derived without considering mesophyll conductance, while mesophyll conductance has been included in the *in vivo* estimates of Walker *et al.* (2013).

An analogous modeling exercise was conducted to compare the potential effects of using the average temperature parameters of C_3_ Rubiscos (taken from the *in vitro* compilation, Tables 2 and 3) and any of the three datasets widely used in photosynthesis modeling (Fig. 8). As with the C_3_ cool *vs*. warm comparison, the differences among simulated *A*_Rubisco_ by different Rubisco temperature responses depended on the temperature range and *C*_c_, but in general, the *in vitro* datasets, i.e. *in vitro* C_3_ average from the present study and that of *S. oleracea* from Jordan and Ogren (1984), yielded higher simulated *A*_Rubisco_ than the *in vivo* datasets based on *N. tabacum* from Bernacchi *et al.* (2001) and Walker *et al.* (2013). Irrespective of *C*_c_, simulated *A*_Rubisco_ of the *in vitro* C_3_ average was similar to simulated *A*_Rubisco_ of *N. tabacum* from Walker *et al.* (2013) at the lower temperature range (<15 ºC), and similar to that of *S. oleracea* from Jordan and Ogren (1984) at the higher temperature range (>40 ºC). Between 15 and 40 ºC, simulated *A*_Rubisco_ of the *in vitro* C_3_ average was lower than that of *S. oleracea* (Jordan and Ogren, 1984) and higher than that of *N. tabacum* (Bernacchi *et al.*, 2001; Walker *et al.*, 2013). At all *C*_c_ values used in the simulations, differences between simulated *A*_Rubisco_ of the *in vitro* C_3_ average and *in vivo* estimates (Bernacchi *et al.*, 2001; Walker *et al.*, 2013) became larger at higher temperatures (Fig. 8, according to paired samples *t*-tests, *P*<0.001 for all these comparisons mentioned above).

The differences in predicted *A*_Rubisco_ temperature relationships from the three datasets of Rubisco kinetics were extensive. For 12 comparisons with temperature response curves derived for four *C*_c_ (120, 150, 200 and 400 μmol mol^−1^), average σ_A_±SE=4.5±0.8 μmol m^−2^ s^−1^ and σ_S_=31±12 μmol m^−2^ s^−1^. These differences were much greater than the differences in predicted *A*_Rubisco_ temperature relationships among cool and warm C_3_ species (Fig. 7 *vs*. Fig. 8; see above for σ_A_ and σ_S_ values), and the differences in predicted *A*_Rubisco_ temperature responses for cool and warm C_3_ species from the *A*_Rubisco_ responses predicted by the mean *in vitro* data for C_3_ species (for these eight comparisons, average σ_A_=0.65±0.18 μmol m^−2^ s^−1^ and average σ_S_=0.93±0.48 μmol m^−2^ s^−1^). Indeed, the average σ_A_ and σ_S_ values for *A*_Rubisco_ predictions using C_3_ *in vitro* kinetics and the average σ_A_ and σ_S_ values for *A*_Rubisco_ predictions using the three selected Rubisco kinetics datasets were significantly different (*P*<0.005 according to pairwise *t*-tests).

## Discussion

### Rubisco *in vitro* temperature relationships: caveats and potentials

To our knowledge, the present study provides the most complete dataset of temperature dependences of Rubisco kinetic characteristics including altogether 17 *in vitro* and seven *in vivo* studies providing information for 49 species from most autotrophic kingdoms of life (Tables 2 and 4). As with any meta-analysis, creating such a synthetic summary inevitably requires analysing the effects of different experimental protocols on the observed trait values. In the case of Rubisco, one should be particularly careful when comparing data from different labs, due to differences in the assay conditions and the calculation of CO_2_ concentration in the assay medium.

In our analysis, we have considered all the assay medium conditions and developed appropriate corrections to standardize for study-to-study differences in assay conditions (Eqs 1–7). In addition to the effects of assay medium composition on the equilibrium CO_2_ concentration, which is known to affect *K*_c_ estimations (Yokota and Kitaoka, 1985, Eqs 1 and 2), we have further highlighted the potential problems with O_2_ solubility that affect *K*_o_ estimation (Eqs 3–6). Furthermore, appropriate corrections were provided for *S*_c/o_ (Eq. 7), which is potentially affected by both bicarbonate equilibrium and O_2_ solubility. Although the corrections of *K*_c_, *K*_o_ and *S*_c/o_ values at any given temperature were large in several cases, the temperature effect on these corrections was relatively weak, such that the absolute trait values at all temperatures were similarly affected. Thus, the overall temperature sensitivity as characterized by the activation energy (∆*H*_a_) and *Q*_10_ values was much less affected by the applied corrections (in most cases <5%).

Although part of the compiled variation may still be due to differences in the methodology from the different reports, we argue that with the corrections applied we have overcome discrepancies in the concentration of CO_2_ and O_2_ in the assay media. Furthermore, data were filtered as explained in the Methods, with the most problematic reports being excluded from the analyses. Analysis of the data also indicates that (i) independent measurements for the same species yielded similar ∆*H*_a_ and *Q*_10_ values (see *S*_c/o_ for *Spinacia oleracea* and *Flaveria pringlei* in Table 2), and (ii) the variability within the main groups of species was low (Tables 2 and 3), suggesting that the approach developed for *a posteriori* standardization of Rubisco characteristics was sound. Thus, we consider that the temperature responses reported here are robust, and that the data in Tables 2 and 3 mainly reflect biological differences.

We note that, although up to seven phylogenetic groups had temperature responses of Rubisco kinetics, data for some phylogenetic groups (in particular Archaea, Proteobacteria, Cyanobacteria and Rhodophyta) were represented by very few species. We therefore suggest that more species of these phylogenetic groups should be surveyed in the future to confirm the trends observed in the present database. Future surveys should include the analysis of the temperature responses of Rubisco kinetics from non-vascular plant groups (liverworts, hornworts and mosses) and ferns, which have not been studied so far. This would allow gaining deeper insight into the evolutionary trends in the temperature response of Rubisco catalysis.

### What do we learn from the compiled dataset? Important species variations in the temperature dependence of Rubisco kinetics suggest adaptation to the thermal environment and photosynthetic mechanism of the species

Among *in vitro* data, *S*_c/o_ (the present study) and $k_{\text{cat}}^{\text{c}}$ (Galmés *et al.*, 2015) are the best documented traits, compared with *K*_c_ and especially *K*_o_, in terms of its temperature response (Table 2). With the exception of the excluded study by Ezaki *et al.* (1999), showing an increase in *S*_c/o_ of *Thermococcus kodakariensis* with increasing temperatures, all compiled reports agree that *S*_c/o_ decreases with increasing temperature, while $k_{\text{cat}}^{\text{c}}$ and *K*_c_ increase (Figs 1 and 2; Galmés *et al.*, 2015).

Despite general trends, the data indicate that the temperature response of Rubisco kinetics varies among phylogenetic groups (Figs 1A and 2A; Galmés *et al.*, 2015). In this sense, Spermatophyta present higher ∆*H*_a_ for *S*_c/o_ (in absolute values) than Proteobacteria and lower values compared with Rhodophyta (Table 3). A similar trend has been observed in the comparative analysis of ∆*H*_a_ for $k_{\text{cat}}^{\text{c}}$ among these phylogenetic groups (Galmés *et al.*, 2015), indicative of adjustment in the temperature sensitivity of Rubisco kinetics. As for *S*_c/o_, this adjustment seems to be related to the *T*_growth_ where the species evolved, with those species from warmer environments presenting a higher thermal dependence of *S*_c/o_ (Fig. 3A).

The lack of correlation between ∆*H*_a_ for $k_{\text{cat}}^{\text{c}}$ and *T*_growth_ apparently contradicts a co-adjustment of Rubisco kinetics to the prevailing environmental conditions (Fig. 3C), and may be attributed to the different set species included in each correlation. Actually, considering all species, the range of variation in ∆*H*_a_ for these two parameters is similar, about 3.5-fold (Table 2 and Galmés *et al.* 2015). Moreover, excluding the data for the cyanobacterium *Thermosynechococcus elongatus*, with vastly different Rubisco kinetics, the results suggest a significant correlation between ∆*H*_a_ for *S*_c/o_ and $k_{\text{cat}}^{\text{c}}$ (Fig. 4). In other words, those Rubiscos with a large increase in $k_{\text{cat}}^{\text{c}}$ with temperature also present a large decrease in *S*_c/o_. This finding may be related to the widely described trade-off between *S*_c/o_ and $k_{\text{cat}}^{\text{c}}$ at 25 ºC (Tcherkez *et al.*, 2006), suggesting that this trade-off is also valid at other temperatures. It should be noted that this correlation was made using average data for the different groups, and that different species were used to obtain the group averages. When the same correlation was analysed for the subset of species for which both *S*_c/o_ and $k_{\text{cat}}^{\text{c}}$ have been measured at different temperatures (nine Spermatophytes), a non-significant relationship was found (data not shown).

Within Spermatophyta, ∆*H*_a_ for *$k_{\text{cat}}^{\text{c}}$* and *K*_c_ of Rubiscos from C_4_ plants was lower compared with C_3_ plants from warm habitats (Table 3), in agreement with recent observations in congeneric species of *Flaveria* with contrasting photosynthetic mechanism (Perdomo *et al.*, 2015). Differences between C_3_ plants from cool and warm habitats were significant in ∆*H*_a_ for *$k_{\text{cat}}^{\text{c}}$*, but not in ∆*H*_a_ for *K*_c_. On average, the ratio between ∆*H*_a_ for $k_{\text{cat}}^{\text{c}}$ and ∆*H*_a_ for *K*_c_ was 1.37, 1.40 and 1.52 for C_3_ plants from cool and warm habitats and C_4_ plants, respectively. This observation indicates that the carboxylase catalytic efficiency ($k_{\text{cat}}^{\text{c}}$/*K*_c_) varies with temperature and suggests that $k_{\text{cat}}^{\text{c}}$/*K*_c_ increases more steeply with temperature in C_4_ plants than in C_3_ plants. Perdomo *et al.* (2015) reported the same trend between C_3_ and C_4_ *Flaveria* species, and related these differences to specific structural changes in the C_4_ Rubisco causing a conformational change that favors product formation (i.e. higher $k_{\text{cat}}^{\text{c}}$) at the expense of weaker substrate affinity (i.e. higher *K*_c_).

In spite of the trend for increased ∆*H*_a_ for *S*_c/o_ (in absolute values) in C_3_ plants from warm habitats as compared with C_3_ plants from cool habitats, differences between these two groups averages were non-significant (Table 3). This result is in agreement with Galmés *et al.* (2005), who reported a tendency for higher temperature dependence for *S*_c/o_ in Mediterranean C_3_ species from warmer habitats, although with some exceptions (e.g. *Lysimachia minoricensis* from wettest and coolest environments displaying also a high activation energy). On the other hand, the non-significant difference in ∆*H*_a_ for *S*_c/o_ between C_3_ and C_4_ plants is in line with the observations of Perdomo *et al.* (2015). Overall, differences in the temperature sensitivity between *S*_c/o_ and $k_{\text{cat}}^{\text{c}}$/*K*_c_ suggest that the temperature dependences for $k_{\text{cat}}^{\text{c}}$/*K*_c_ and *k*_cat_^o^/*K*_o_ also differ among C_3_ and C_4_ plants.

Given the observed variability, we suggest using the corrected species-specific functions describing the temperature dependence of Rubisco kinetic parameters (shown in Table 2) to model photosynthesis in the different species. If the temperature response of Rubisco kinetic parameters has not been described for the species under study, we recommend using the groups’ average values (presented in Table 3) to increase the accuracy of photosynthesis models in the different functional groups.

### The temperature dependence of *K*_o_: apparent contradictions among *in vitro* data

The temperature response of *K*_o_ based on *in vitro* data is available for five Spermatophyta species (Table 2), with contradictory results. A quantitatively similar value of ∆*H*_a_ for *K*_o_ to those of ∆*H*_a_ for *K*_c_ in Spermatophyta has been reported only for *Atriplex glabriuscula* (Badger and Collatz, 1977). The overall difficulty with *K*_o_ estimations is that Rubisco is much less specific for O_2_ than for CO_2_, and the oxygenase reaction of Rubisco is not saturated even in a 100% O_2_ atmosphere. Estimation of *K*_o_ over an O_2_ concentration range exhibiting only a slight non-linearity is extremely sensitive to any errors in gas concentrations. According to the compiled evidence, the temperature dependence of *K*_o_ for the other three species is very low compared with that for *K*_c_, with values of ∆*H*_a_ for *K*_o_ close to zero, or even negative for *Glycine max* (Laing *et al.*, 1974) and *Setaria viridis* (Boyd *et al.*, 2015). Apparently, this discrepancy is not due to the specific methods used by the different studies as qualitatively different temperature scaling functions have been observed using the same methodologies, and similar responses with different methodologies: Badger and Collatz (1977) and Lehnherr *et al.* (1985) measured oxygenase activity using an oxygen electrode, Jordan and Ogren (1984) obtained *K*_o_ from the inhibition of carboxylase activity at 50% O_2_ (i.e. *K*_c,app_), Laing *et al.* (1974) measured the oxygenase activity at different O_2_ concentrations by spectrophotometric determination of a P-glycolate derivate, and Boyd *et al.* (2015) used membrane inlet mass spectrometry to measure the temperature responses of Rubisco kinetics.

### Common trends and discrepancies between *in vitro* and *in vivo*-based data on the temperature dependence of Rubisco kinetics

*In vivo* data on the temperature response of *K*_o_ agree in that values of ∆*H*_a_ for *K*_o_ are positive (i.e. *K*_o_ increasing with assay temperature, Fig. 5C), although with lower values compared with the *in vitro*-based trend described for *Atriplex glabriuscula* (Badger and Collatz, 1977) (Tables 2 and 4). The comparison of the *in vitro* and *in vivo* measurements of the temperature response of *K*_o_ in *Glycine max* exemplifies how the discrepancies between *in vitro* and *in vivo* are particularly important for this kinetic parameter (Fig. 6E). Presence of some CO_2_ is inevitable in *in vivo* measurements due to mitochondrial respiration, which could be affected by [O_2_] itself. Typically, only two, seldom three, O_2_ concentrations have been used in *K*_o_ estimations, but given the high sensitivity of these measurements to O_2_ concentration, we argue that both *in vivo* and *in vitro* measurements of *K*_o_ can be improved by using more O_2_ concentrations in the assays. Overall, *K*_o_ (or the Michaelis–Menten constant of Rubisco for CO_2_ at atmospheric conditions, $K_{\text{c}}^{\text{air}}$) is needed to model photosynthesis responses to temperature, and therefore, more *in vitro* and *in vivo* data on the temperature dependence of *K*_o_ are required in a larger number of species to confirm observed trends and solve discrepancies.

Contrary to *K*_o_, *in vitro* and *in vivo* determinations of the temperature response of *S*_c/o_ and *K*_c_ describe similar shapes, with the *in vitro* averages lying intermediate between *in vivo* estimates (Fig. 5). In this case, methodological issues associated with *in vitro* assays and *in vivo* estimations may determine the degree of agreement between *in vitro* and *in vivo* reports. For instance, *in vitro* and *in vivo* data on the temperature response of *S*_c/o_ in *Spinacia oleracea* match very well between 15 and 45 ºC (Fig. 6A), with minor differences in the ∆*H*_a_ (Tables 2 and 4). The values of ∆*H*_a_ for *S*_c/o_ in *Triticum aestivum* are also similar between *in vitro* (−19.7 kJ mol^−1^) and *in vivo* (−25.2 kJ mol^−1^), although absolute values of *in vitro S*_c/o_ are approximately 1.6-fold higher than *in vivo* ones across the range of measurement temperatures (Fig. 6B).

Both *in vitro* and *in vivo* data on Rubisco kinetics present concerns implicitly linked to the specific methodologies. Since Monson *et al.* (1982), the first study discussing the possible reasons for discrepancies between *in vitro* and *in vivo* data, other studies have added to the debate on whether *in vitro* or *in vivo* data are more reliable and should be used in photosynthesis models (Ghashghaie and Cornic, 1994; von Caemmerer *et al.*, 1994; Rogers *et al.*, 2001; Galmés *et al.*, 2011*b*; Díaz-Espejo, 2013; Walker *et al.*, 2013). The main concerns related to *in vitro* measurements are that assay conditions may not be representative of the solute-rich environment of a chloroplast stroma where Rubisco operates, and that the extraction may not be fully efficient in terms of enzyme recovery and activation (Monson *et al.*, 1982; Rogers *et al.*, 2001; Galmés *et al.*, 2011*b*; Díaz-Espejo, 2013). The latter, however, will have no consequences for *S*_c/o_, *K*_c_ and *K*_o_ assays since they are independent of Rubisco concentration. Furthermore, as we have observed, the differences in assay conditions have only a moderate effect on ∆*H*_a_ and *Q*_10_ values. In turn, difficulties in the *in vivo* estimations are mainly related to the quantification of the actual concentration of CO_2_ at the site of carboxylation (*C*_c_) and the rate of mitochondrial respiration in the light (*R*_light_), in addition to the concerns associated with the leaf mesophyll heterogeneity in terms of photosynthetic capacity, CO_2_ concentration and light reaching the chloroplasts (Ghashghaie and Cornic, 1994; von Caemmerer *et al.*, 1994; Díaz-Espejo, 2013; Walker *et al.*, 2013). The accurate quantification of *C*_c_ depends on the estimation of the leaf mesophyll conductance (*g*_m_), which relies on a series of assumptions (see for instance Tholen *et al.*, 2012; Busch *et al.*, 2013; Loriaux *et al.*, 2013; Gu and Sun, 2014). The incorporation of *g*_m_ in photosynthesis models has resulted in an improvement of the *in vivo* estimates of Rubisco kinetics and partially reconciled *in vitro* and *in vivo* data (von Caemmerer *et al.*, 1994; Walker *et al.*, 2013). In addition to technical difficulties in both *in vitro* and *in vivo* measurements, we note that there has not been much attention on how to conduct valid comparisons among *in vivo* and *in vitro* data. As our methodological analysis demonstrates, the comparison is not trivial because the conversion of kinetic characteristics among gas (*in vivo* data) and liquid (*in vitro* data) phases is strongly dependent on equilibrium constants used (Eqs 8–10).

A principal problem with *in vivo* measurements is that derivation of Rubisco kinetics using inverse modeling techniques becomes increasingly challenging at the physiological limits, e.g. leaf temperatures above 35–40 °C and below 10–15 °C, and especially in conditions leading to heat (above *ca* 45 °C) and cold stress (below *ca* 5 °C). Under higher temperatures, enhanced mitochondrial respiration, inactivation of Rubisco, closure of stomata and unclear response of *g*_m_ preclude any accuracy of derivation of Rubisco kinetics (Crafts-Brandner and Salvucci, 2000; Atkin and Tjoelker, 2003; von Caemmerer and Evans, 2015). At lower temperatures, the challenges are associated with overall low gas-exchange fluxes due to low stomatal conductance and enzymatic capacities and again unclear effects of *g*_m_ (Ensminger *et al.*, 2012). Even if *g*_m_, mitochondrial and stomatal effects could be accounted for, the principal problem still remaining is that Rubisco-limited photosynthesis under such conditions is driven by combined effects of Rubisco activase and Rubisco. In the case of heat and cold stress conditions, the situation is further complicated because of time-dependent reductions in enzymatic capacities (Niinemets and Keenan, 2014). On the other hand, the problems associated with *in vitro* assays at low and high temperatures can originate from low reaction rates and changes in the substrate concentration in the assay media due to evaporation, respectively, which may increase the error associated with these measurements. It may well be that the lower *Q*_10_ values for *K*_c_ at higher temperatures observed for *in vivo* data compared with *in vitro* data (Fig. 5B) reflect the outlined problems with measurements at higher temperature. We suggest that both *in vivo* and *in vitro* measurements provide informative insight into the potential and actual *in vivo* kinetics of Rubisco and we call for more comparative measurements of *in vivo* and *in vitro* Rubisco kinetics in economically and ecologically relevant species.

### Modeling Rubisco-dependent photosynthesis: how much do all the observed differences in temperature kinetics matter?

Although statistically important differences were observed in a number of Rubisco kinetic characteristics determined in *in vitro* studies (Table 3), the differences were relatively small, and the key question is how much do these differences matter when considered together? A comparison of simulated Rubisco-dependent photosynthesis among C_3_ cool and warm species demonstrates that the temperature adaptation indeed improves modeled photosynthesis of C_3_ cool species under lower temperatures, especially at lower chloroplastic CO_2_ concentration, while the temperature adaptation increases modeled photosynthesis of C_3_ warm species under higher temperatures, especially at higher chloroplastic CO_2_ concentration (Fig. 7). This is an important outcome, suggesting that in addition to higher-level cellular, whole leaf and whole plant adaptation responses (e.g. Björkman *et al.*, 1980; Silim *et al.*, 2010; Muhl *et al.*, 2011), enzyme-level adaptation can lead to significant modifications in realized leaf photosynthesis in plants adapted to different climates. Of course, it is only a simulation for two groups of species that have relatively similar Rubisco characteristics and only considers the potential Rubisco-limited photosynthesis. Nevertheless, across all species, the activation energy for *S*_c/o_ (Fig. 3A) and the deactivation energy for *k*_cat_ scaled with the growth temperature (Galmés *et al.*, 2015), indicating that the simulations shown for C_3_ cool and warm species constitute a part of a broad trend of Rubisco temperature acclimation. Combining temperature responses of Rubisco-limited and RuBP-regeneration-limited photosynthesis further indicates that Rubisco-limited photosynthesis is a key driver of realized photosynthesis over much of the ambient temperature response for a large fraction of physiologically relevant quantum flux densities and chloroplastic CO_2_ concentrations (Galmés *et al.* 2014*a*).

The difference among C_3_ cool and warm plants was predicted to increase with increasing CO_2_ concentration (Fig. 7). This difference in Rubisco temperature characteristics is expected to enhance the competitive potential of C_3_ warm species relative to C_3_ cool species in future climates with higher atmospheric CO_2_ concentration and air temperature (Kirtman *et al.*, 2013). We believe that for realistic simulation of carbon gain in future climates, models of C_3_ photosynthesis need to be modified to incorporate different Rubisco temperature kinetics of broad species groups to reflect modifications in Rubisco temperature kinetics to species’ growth environment. Even by doing this, we still need to recognize the inherent variability in species’ Rubisco temperature responses within species groups that we cannot explain by species’ growth environment or by phylogenetic relationships (Fig. 3). Such a variability is of particular significance when species group averages need to be used for simulation of photosynthesis for any given species lacking measured Rubisco kinetic traits (or for derivation of FvCB photosynthesis model parameters). However, it is likely of less concern for simulating photosynthesis of multi-species canopies where species-specific effects average out and average kinetics for species groups are more appropriate.

In fact, the problem of selecting ‘the appropriate’ set of kinetic parameters for modeling C_3_ photosynthesis has a long history starting with the publication of the FvCB photosynthesis model (Farquhar *et al.*, 1980). Early model applications used *in vitro* measurements for *Atriplex glabriuscula* (Badger and Collatz, 1977) or *Spinacia oleracea* (Jordan and Ogren, 1984), while today researchers increasingly use the *in vivo* dataset based on *N. tabacum* (Bernacchi *et al.*, 2001). While further *in vivo* datasets are becoming available (Walker *et al.*, 2013), *in vitro* parameters from either Badger and Collatz (1977) or Jordan and Ogren (1984) remain widely used today. In fact, according to Thomson Reuters Web of Science (accessed 3 May 2016), Farquhar *et al.* (1980), the paper with the FvCB model, has been cited 3563 times (232 times in 2015), while Bernacchi *et al.* (2001) has been cited 442 times (42 times in 2015) (see also Niinemets *et al.* (2015) for an analysis of the frequency of use of different Rubisco kinetics for a subset of studies using the FvCB model in simulating canopy photosynthesis). The comparison of the three datasets, *in vitro* Jordan and Ogren (1984), *in vivo* Bernacchi *et al.* (2001) and *in vivo* Walker *et al.* (2013), with the *in vitro* C_3_ average dataset derived in our study demonstrated significant differences in predicted Rubisco-limited photosynthesis (Fig. 8). Further, the variability among the photosynthesis estimates derived by the three kinetics datasets is much greater than the variability among the estimated photosynthesis derived by the *in vitro* average kinetics for C_3_ cool and warm datasets developed in the current study.

A detailed examination of differences among model estimates indicated that both *in vivo* datasets provided lower estimates of Rubisco-limited photosynthesis than the C_3_-average and Jordan and Ogren (1984) *in vitro* datasets (Fig. 8). This is an important difference for interpretation of the FvCB model’s parameters, in particular *V*_cmax_, the maximum carboxylase activity of Rubisco, derived from gas-exchange data (Rogers, 2014). Use of the *in vivo* kinetics of Bernacchi *et al.* (2001) to derive *V*_cmax_ from net assimilation *vs.* CO_2_ response curves leads to much higher, even more than 50% higher, estimates of *V*_cmax_ at 25 °C than use of the *in vitro* Jordan and Ogren (1984) kinetics. This difference is not fully appreciated by the modeling community, especially by modelers working at canopy, landscape, regional and global levels. Obviously, part of the large difference of the *in vitro* Jordan and Ogren (1984) and *in vivo* Bernacchi *et al.* (2001) kinetics could reflect lack of mesophyll diffusion conductance in parameter estimation in the study of Bernacchi *et al.* (2001), especially given the important temperature effects on mesophyll conductance (Bernacchi *et al.*, 2002). This is partly confirmed by the smaller differences in *in vitro* and *in vivo* estimates from the dataset of Walker *et al.* (2013) that is derived considering mesophyll conductance. Nevertheless, as our study demonstrates, there are species-specific differences in Rubisco temperature kinetics, and this comparison again emphasizes the principal problem of using a single Rubisco temperature kinetics to simulate photosynthesis of all C_3_ species. Researchers should be fully aware about the limitations and associated uncertainties of using a single Rubisco temperature dataset.

### Concluding remarks

The purpose of our study was two-fold: to summarize all the existing information on Rubisco temperature kinetics and analyse the relationships between Rubisco temperature kinetics within and among the phylogenetic and functional groups. We have standardized all the available *in vitro* Rubisco temperature data and constructed an extensive database that allows for direct comparison of Rubisco temperature kinetics without possible study-to-study differences due to assay buffer composition. We believe that in addition to identification of what is available, a key strength of this analysis is recognition of the gaps in data coverage. Too often relevant research is not conducted because there is a feeling in the community that ‘all this has already been measured’. As our analysis demonstrates, at least concerning Rubisco temperature kinetics, this opinion is an illusion and the coverage of several species groups is poor, suggesting that there clearly is room for high quality Rubisco temperature kinetic measurements over the years to come. This new research could potentially challenge some of the relationships developed here.

The other widespread opinion is that *in vitro* data coming from different labs and obtained using somewhat different methodologies cannot be used for broad analyses. We believe that the standardization functions developed by us largely solve this issue. Furthermore, we observed that activation energies and *Q*_10_ values that describe the magnitude of temperature-dependent change of given Rubisco kinetics are not very sensitive to the corrections developed, suggesting that our analysis of Rubisco temperature trait covariation is robust. However, we strongly advise against uncritical use of data by simply pooling the information without paying due attention to the experimental details.

From an immediate practical perspective, we provide separate average Rubisco temperature kinetics for C_3_ species from cool and warm habitats and argue that use of separate kinetics is warranted in models of carbon gain, especially for simulation of future conditions. Comparison of Rubisco temperature kinetics widely used so far further suggests that the modeling community needs to rethink the concept of ‘single species Rubisco fits all’. In fact, the variability in photosynthesis predictions from widely used single species datasets was much bigger than the variability between photosynthesis predictions by C_3_ cool and warm datasets developed here. Although we acknowledge the inertia in the modeling community, at least uncertainties of using single species models need consideration in simulating photosynthesis from leaf to globe.
